# Liver Cancer Mortality Disparities at a Fine Scale Among Subpopulations in China: Nationwide Analysis of Spatial and Temporal Trends

**DOI:** 10.2196/54967

**Published:** 2024-08-08

**Authors:** Ting Gan, Yunning Liu, Hilary Bambrick, Maigeng Zhou, Wenbiao Hu

**Affiliations:** 1 Ecosystem Change and Population Health Research Group, School of Public Health and Social Work, Queensland University of Technology, Brisbane, Australia; 2 National Center for Chronic and Noncommunicable Disease Control and Prevention, Chinese Center for Disease Control and Prevention, Beijing, China; 3 National Centre for Epidemiology and Population Health, Australian National University, Canberra, Australia

**Keywords:** liver cancer, mortality, year of life lost, spatial distribution, temporal trend

## Abstract

**Background:**

China has the highest number of liver cancers worldwide, and liver cancer is at the forefront of all cancers in China. However, current research on liver cancer in China primarily relies on extrapolated data or relatively lagging data, with limited focus on subregions and specific population groups.

**Objective:**

The purpose of this study is to identify geographic disparities in liver cancer by exploring the spatial and temporal trends of liver cancer mortality and the years of life lost (YLL) caused by it within distinct geographical regions, climate zones, and population groups in China.

**Methods:**

Data from the National Death Surveillance System between 2013 and 2020 were used to calculate the age-standardized mortality rate of liver cancer (LASMR) and YLL from liver cancer in China. The spatial distribution and temporal trends of liver cancer were analyzed in subgroups by sex, age, region, and climate classification. Estimated annual percentage change was used to describe liver cancer trends in various regions, and partial correlation was applied to explore associations between LASMR and latitude.

**Results:**

In China, the average LASMR decreased from 28.79 in 2013 to 26.38 per 100,000 in 2020 among men and 11.09 to 9.83 per 100,000 among women. This decline in mortality was consistent across all age groups. Geographically, Guangxi had the highest LASMR for men in China, with a rate of 50.15 per 100,000, while for women, it was Heilongjiang, with a rate of 16.64 per 100,000. Within these regions, the LASMR among men in most parts of Guangxi ranged from 32.32 to 74.98 per 100,000, whereas the LASMR among women in the majority of Heilongjiang ranged from 13.72 to 21.86 per 100,000. The trend of LASMR varied among regions. For both men and women, Guizhou showed an increasing trend in LASMR from 2013 to 2020, with estimated annual percentage changes ranging from 10.05% to 29.07% and from 10.09% to 21.71%, respectively. Both men and women observed an increase in LASMR with increasing latitude below the 40th parallel. However, overall, LASMR in men was positively correlated with latitude (*R*=0.225; *P*<.001), while in women, it showed a negative correlation (*R*=0.083; *P*=.04). High LASMR areas among men aligned with subtropical zones, like Cwa and Cfa. The age group 65 years and older, the southern region, and the Cwa climate zone had the highest YLL rates at 4850.50, 495.50, and 440.17 per 100,000, respectively. However, the overall trends in these groups showed a decline over the period.

**Conclusions:**

Despite the declining overall trend of liver cancer in China, there are still marked disparities between regions and populations. Future prevention and control should focus on high-risk regions and populations to further reduce the burden of liver cancer in China.

## Introduction

Liver cancer remains a significant public health concern. Of all cancers, the disability-adjusted life years (DALYs) of liver cancer ranks fifth highest in health burden globally, with approximately 12.5 million DALYs [1]. While the global incidence and mortality of liver cancer remained relatively stable from 2010 to 2019, the sharp surge in new cases and deaths underscores the undeniable impact of liver cancer [1]. The International Agency for Research on Cancer predicted that, compared to 2020, the new cases and deaths of liver cancer will increase by 55% and 56% by 2040, respectively [2]. The growth of liver cancer burden is particularly evident in some high-income countries. For example, liver cancer incidence in the United States steadily rose from 5.0 in 2000 to 8.7 per 100,000 in 2019 [3]. In the United Kingdom, liver cancer incidence and mortality increased by approximately 54% and 63% from 2007 to 2017 [4]. Similarly, Australia has faced such a dramatic increase in the past decades [5]. However, in China, liver cancer has generally shown a more optimistic trend but varied by region.

Liver cancer has been well controlled in China, with appreciations to the national hepatitis B virus immunization program over the past 20 years, but it remains at the forefront of all cancers in China [6]. However, China has the highest number of liver cancers worldwide, contributing nearly 42.5% of DALYs for liver cancer worldwide [6]. The unusual upward trend in high-income countries makes it seem premature to celebrate China’s recent decline in liver cancer, given that the country is currently in a period of rapid development. Meanwhile, China’s vast geographical area and diverse social lifestyles may lead to variations in the distribution of liver cancer, which implies that the recent positive national picture of liver cancer cannot be uniform across all regions and populations. Nationwide, liver cancer mortality in China has shown a decreasing trend, but regional differences persist, with higher rates in rural areas compared to urban areas [7]. In some developed regions, such as Shanghai, this decreasing trend is significant [8], while in regions such as Yunnan and Nei Mongolia, an upward trend has been observed [9,10]. Although these related studies have described the trend or distribution of liver cancer in China to varying degrees, many of them only focus on a specific region or merely on its trends, failing to provide a comprehensive overview of liver cancer mortality across the entire country.

Furthermore, our earlier study on liver cancer in Australia found the distribution of liver cancer across different climate zones, which suggested that there may be climatic zone–related disparities in liver cancer [11]. This result implied that liver cancer may be affected by climate change. Some studies have suggested that climate change may increase the risk of skin cancer through ultraviolet exposure or rising temperatures [12], and for other cancers, such as lung cancer, temperature or extreme weather may also exacerbate the risk of death from air pollution [13]. Indeed, climate change can affect the occurrence and development of cancer through multiple pathways. Whether by increasing the risk of exposure to carcinogens or by affecting the accessibility of health care facilities, both may have irreversible consequences to some extent [14].

However, in China, there is currently no study describing the distribution of liver cancer by climate zones. In addition, current research on liver cancer in China primarily relies on extrapolated data or relatively lagging data, with limited focus on subregions and specific population groups. Therefore, there is an urgent requirement to identify sensitive or vulnerable areas for liver cancer based on more detailed and updated data. This would provide a critical foundation for subsequent adjustments to liver cancer prevention and control strategies within the population.

This study explored the spatial and temporal patterns and trends of liver cancer mortality and burden in China, using data from the National Death Surveillance System (NDSS), from 2013 to 2020. By defining the gender-specific and age-specific distribution of liver cancer in China, any potential high-risk subregions and subpopulations may be identified.

## Methods

### Data Source and Study Design

Data on liver cancer mortality from January 1, 2013, to December 31, 2020, were obtained from the NDSS. This time period was chosen because the NDSS has been significantly improved since 2013, covering all 31 provinces, autonomous regions, and municipalities in mainland China. Through multilevel hierarchical cluster sampling, after several rounds of representative evaluation and adjustment, the system has included 605 Death Surveillance Points (DSPs) at the county level nationwide, covering 24.3% (n=323,773,287) of the population in Mainland China. Physicians, local Centers for Disease Control and Prevention, and appropriate personnel are required to be trained to report all deaths in the DSP in real time. Centers for Disease Control and Prevention at all levels (ie, provincial, municipal, and county levels) check the completeness, coding accuracy, and logical consistency of the reported data. Any inaccuracies in reporting are rectified by reviewing medical records or performing verbal autopsies, which involve interviews and questionnaires to determine the cause of death. In addition, to address the possibility of underreporting bias, national-level periodic surveys are carried out every 3 years, encompassing a nationally representative sample from all DSPs. The detailed purpose and characteristics of the NDSS have been described previously [15]. Liver cancer is defined as C22 according to the *International Classification of Diseases, Tenth Revision* (*ICD-10*). The DSP data have been widely used in policy making of disease prevention and control in China.

Population data from 2013 to 2020 in mainland China are sourced from the National Bureau of Statistics. To calculate age-standardized mortality rate of liver cancer (LASMR), we used the 2010 census population as a reference for age adjustment. Considering the lag in the effect of hepatitis B on liver cancer and the availability of data, the average incidence of hepatitis B in each province of mainland China from 2004 to 2011 was used as the incidence of hepatitis B in each DSP region. The hepatitis B data were collected from the Data Centre for Public Health Sciences.

In this study, liver cancer mortality and years of life lost (YLL) were described in terms of gender, age, climate classification, and geographical region. Based on the Global Burden of Disease 2019 data in Asia, the DALYs caused by cancer remain relatively low before the age of 35 years, then significantly increase until 50 years, stabilize and peak at 65 years, and then begin to decline [16]. Therefore, age was classified into 4 groups: <35, 35‐49, 50‐64, and 65+ years. Geographical regions were classified into 7 regions according to the criteria of the National Bureau of Statistics: north, northeast, east, central, south, southwest, and northwest. According to the Qinling Mountains-Huaihe River line [17], we classified the 7 regions into 2 parts: the northern part (including the north, northeast, and northwest) and the southern part (including east, central, south, and southwest). Additionally, because the burden of disease may vary by climate, we explored liver cancer distribution and trends across various climate zones [18]. Climate zones were determined based on the Köppen-Geiger classification [19], there are 11 climate zones in China: Am, Aw, BWk, BSk, Cwa, Cwb, Cfa, Dwa, Dwb, Dwc, and ET, covering climates ranging from tropical to polar. Among all 11 climate zones, Am and Aw belong to the tropical region, BWk and BSk belong to the arid region, Cwa, Cwb, and Cfa belong to the subtropical region, Dwa and Dwb belong to the continental region, Dwc belongs to the subarctic region, and ET belongs to the polar region (Multimedia Appendix 1).

### Ethical Considerations

The study was approved by the University Human Research Ethics Committee of Queensland University of Technology (approval 5913, LR 2023-5913-13703). The requirement for informed consent was waived because the data were aggregated to minimize the risk of reidentification.

### Statistical Analysis

The average LASMR from 2013 to 2020 was calculated by gender, age group, and climate classification. We also calculated the year-specific age-standardized mortality. To estimate liver cancer mortality on a continuous spatial field, sex-specific, age-specific, and year-specific average LASMR were interpolated, applying the Ordinary Kriging method, which is widely used for spatial interpolation of diseases to address areal bias and smooth data including DSP data [20,21]. Using the zonal function, we transformed the continuous spatial field estimated by the Ordinary Kriging method into polygons at the provincial level to obtain the average LASMR of 31 provinces (including autonomous regions and municipalities) in Mainland China from 2013 to 2020, and then, liver cancer mortality of each province was ranked. To assess the burden caused by liver cancer, YLLs were calculated from 2013 to 2020, as YLLs are the main contributor to DALYs for liver cancer, with a contribution of 99% [1].

Joinpoint regression was applied to explore the long-term trend of LASMR from 2013 to 2020. This model used the Monte-Carlo permutation test to optimize model fitting for each consecutive linear segment divided from a long-term trend. Average annual percentage changes (AAPCs) and 95% CIs were estimated by joinpoint regression to describe the national LASMR change during the period. AAPC>0 indicated an increasing trend in the national LASMR from 2013 to 2020, while AAPC<0 indicated a decreasing trend for the whole country.

To quantify the geographical distribution of liver cancer mortality changes in Mainland China from 2013 to 2020, we calculated the estimated annual percentage change (EAPC). EAPC>0 indicated an increasing trend in liver cancer mortality in the region from 2013 to 2020, while EAPC<0 indicated a decreasing trend.

Spatial dispersion and trends in liver cancer were assessed by the correlation between latitude and LASMR. This analysis explored a potential association between liver cancer and climate, given the latitudinal zonal distribution pattern of climate. To evaluate this correlation, we used partial correlation [22], accounting for hepatitis B incidence as a controlling variable. Additionally, we conducted seasonal decomposition using locally estimated scatterplot smoothing to assess the monthly patterns of LASMR within distinct climate classifications.

The Kriging interpolation, zonal function, and maps were conducted in ArcGIS Pro (version 3.1.2; Esri, Inc). Joinpoint regression analysis was developed by the Joinpoint Regression Program (version 5.0.2; National Cancer Institute). Other statistical analysis was performed by STATA/MP (version 16.0; StataCorp LLC) and R (version 4.2.3; The R Foundation).

## Results

### Overview

From 2013 to 2020, the number of liver cancer deaths in China reported by the DSP system was 447,392 cases in men and 160,490 cases in women. Deaths in men far exceed those in women, at approximately 2.58 times higher (Table 1). The LASMR was also higher in men over the time. For men, the average LASMR increased from 28.79 (95% CI 27.36-30.22) in 2013 to 32.57 (95% CI 31.07-34.07) per 100,000 in 2015, with an AAPC of 6.54% (Multimedia Appendix 2), and then began to decline, with average LASMR 26.38 (95% CI 25.34-27.43) per 100,000 by 2020. Women experienced a similar pattern, with average LASMR rising from 11.09 (95% CI 10.57-11.62) per 100,000 in 2013 to a peak in 2015, then steadily decreasing to 9.83 (95% CI 9.44-10.23) per 100,000 in 2020. But the overall trend in men remained relatively stable (AAPC −1.38%; 95% CI −2.96% to 0.23%; *P*=.09), while the general trend in women was decreasing (AAPC −1.94%; 95% CI −3.08% to −0.79%; *P*=.001; Table 1).

**Table 1.** Estimated death and average age-standardized mortality rate (1/100,000) of liver cancer in China, 2013‐2020.

	Men		Women	
	Death (n=447,392), n	Average age-standardized mortality rate (1/100,000) (95% CI)	Death (n=160,490), n	Average age-standardized mortality rate (1/100,000) (95% CI)
2013	50,018	28.79 (27.36 to 30.22)	17,703	11.09 (10.57 to 11.62)
2014	57,178	31.79 (30.37 to 33.21)	20,086	12.02 (11.53 to 12.51)
2015	58,154	32.57 (31.07 to 34.07)	20,632	12.17 (11.68 to 12.65)
2016	57,394	31.16 (29.76 to 32.55)	20,340	11.42 (10.96 to 11.87)
2017	57,240	30.98 (29.72 to 32.24)	20,457	11.28 (10.84 to 11.71)
2018	55,853	29.11 (27.34 to 30.28)	20,365	10.74 (10.34 to 11.15)
2019	55,370	27.36 (26.22 to 28.49)	19,973	10.17 (9.77 to 10.56)
2020	56,185	26.38 (25.34 to 27.43)	20,934	9.83 (9.44 to 10.23)
AAPC^a^ (%) (95% CI)	—^b^	−2.06 (−4.15 to 0.07)	—	−2.42 (−4.54 to −0.26)

a AAPC: average annual percentage change.

b Not applicable.

### Spatial Distribution

**Figure 1.**
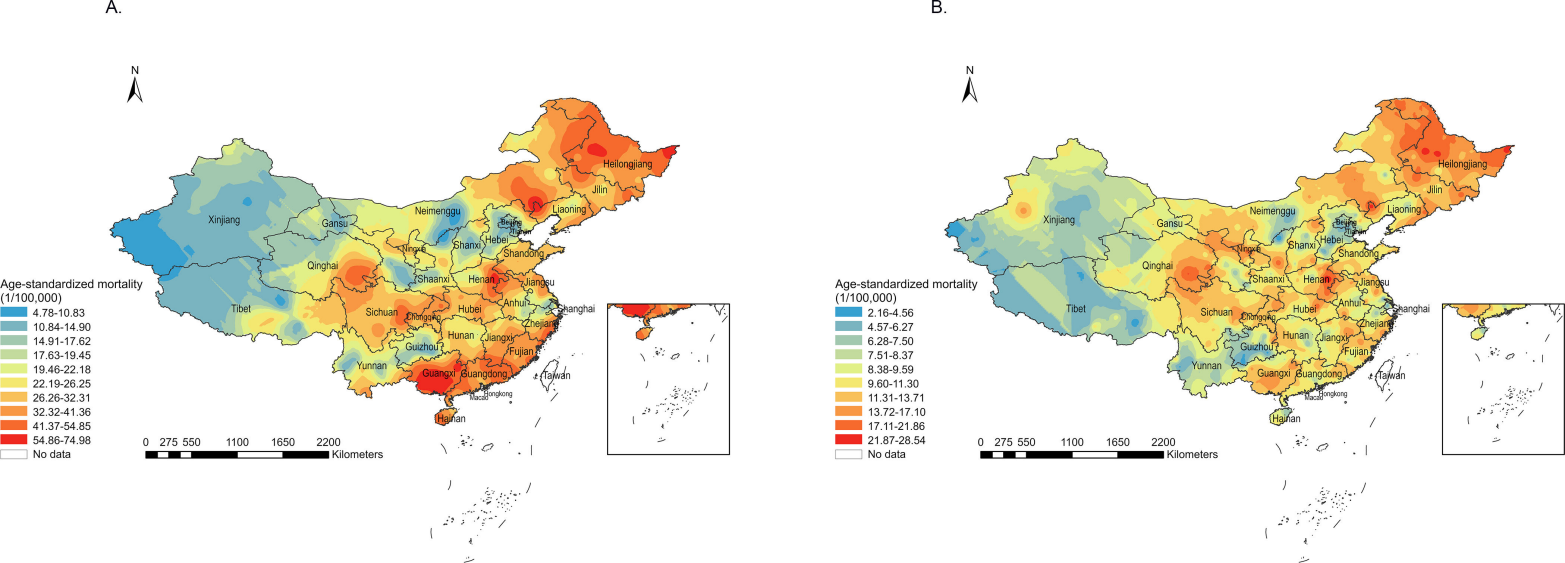
Spatial distribution of age-standardized liver cancer mortality rate among men (**A**) and women (**B**) in China, 2013‐2020.

Figure 1 shows the spatial distribution of average LASMRs conducted by Ordinary Kriging interpolation from 2013 to 2020 by gender in China. The spatial patterns show diversity between men and women. The regions with the highest LASMRs among men are mainly in the south, southeast, and northeast, especially in Guangxi Province, where LASMRs ranged from 54.86 to 74.98 per 100,000 in numerous counties of this province, and in Heilongjiang Province, where LASMRs ranged from 32.32 to 74.98 per 100,000 in most counties. However, the higher LASMRs were relatively scattered in other regions. Among women, there were no significant clusters of high LASMRs in the south as men. The regions with higher average LASMRs from 2013 to 2020 were predominantly clustered in the northeast in women, especially in Heilongjiang and Jilin, with LASMRs ranging from 13.72 to 21.86 per 100,000 in most counties.

Of the 31 provinces in China, Guangxi appeared the highest average LSAMR, with a LASMR of 50.15 per 100,000, followed by Heilongjiang, Guangdong, Hainan, and Fujian. Among the top 5 LASMRs in men, most were located in the southern part, and only 1 in the northern part. In contrast, provinces with higher LASMRs were mainly situated in the northern part in women, with the highest LASMR observed in Heilongjiang at 16.64 per 100,000. Although the ranking was inconsistent, the provinces with the lowest LASMRs for both women and men were Xinjiang, Beijing, Tianjin, Tibet, and Guizhou (Table 2).

**Table 2.** Estimated average liver cancer mortality rankings of provinces by gender in China, 2013‐2020.

Men		Women	
Province	Average age-standardized mortality (1/100,000)	Province	Average age-standardized mortality (1/100,000)
Guangxi	50.15	Heilongjiang	16.64
Heilongjiang	43.36	Jilin	13.75
Guangdong	41.93	Henan	13.20
Hainan	39.25	Ningxia	12.88
Fujian	38.42	Chongqing	12.61
Chongqing	34.88	Neimenggu	11.99
Jilin	34.19	Guangxi	11.99
Jiangxi	33.14	Sichuan	11.70
Henan	32.89	Liaoning	11.68
Sichuan	31.62	Fujian	11.62
Zhejiang	31.45	Hubei	11.42
Liaoning	31.19	Anhui	11.00
Hubei	30.56	Shandong	10.95
Shandong	29.69	Jiangsu	10.95
Anhui	28.87	Zhejiang	10.84
Jiangsu	28.43	Jiangxi	10.78
Neimenggu	28.40	Shananxi	10.76
Hunan	27.42	Guangdong	10.70
Ningxia	24.27	Qinghai	10.64
Yunnan	22.92	Gansu	10.51
Qinghai	22.65	Shanxi	10.50
Shananxi	21.14	Hunan	10.21
Hebei	20.80	Hainan	8.85
Shanghai	20.54	Hebei	8.70
Gansu	19.78	Yunnan	8.05
Shanxi	19.65	Shanghai	7.78
Guizhou	19.57	Xinjiang	7.66
Tibet	17.14	Guizhou	7.64
Tianjin	15.32	Tibet	6.97
Beijing	15.15	Beijing	6.78
Xinjiang	13.21	Tianjin	6.62

### Spatial and Temporal Variation

As shown in Multimedia Appendix 3, the distribution of LASMRs in China varied over time. From 2013 to 2016, the regions with high LASMRs among men demonstrated a broader geographical spread, mainly encompassing the south and northeast. During this period, the LASMRs ranged from 4.67 to 57.01 per 100,000. The situation changed slightly from 2017 to 2020, with Guangxi remaining a high LASMR area, while Sichuan, Jiangsu, and Zhejiang had significantly fewer high mortality areas. Among women, high LASMR areas were still mainly concentrated in Heilongjiang from 2017 to 2020, with a decrease in high LASMR areas in Guangdong, Fujian, and Zhejiang compared to 2013 to 2016.

**Figure 2.**
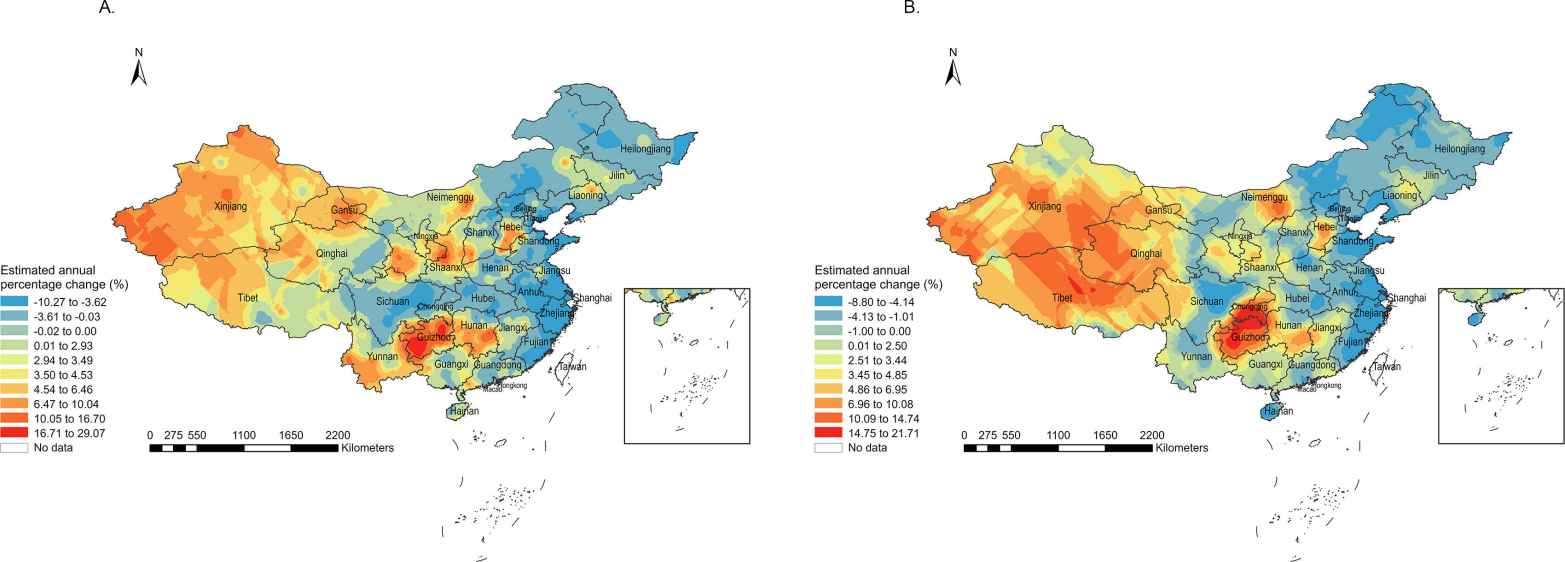
Estimated annual percentage change from 2013 to 2020 of age-standardized liver cancer mortality rate among men (**A**) and women (**B**) in China.

From 2013 to 2020, there was a noticeable variation in the EAPC of LASMR distribution in China compared to the average LASMR distribution (Figure 2). Guizhou was the province with the most pronounced increase in LASMRs among men, with an EAPC between 10.05% and 29.07%. Among women, along with Guizhou, Chongqing also experienced a significant increase in LASMRs. The increases in LASMRs for women in Chongqing ranged from 10.09% to 21.71% in most regions. In addition, among both men and women, many areas in the northwest also showed an increasing trend, but the DSPs in this region are more sparse.

### Age-Specific Mortality

**Figure 3.**
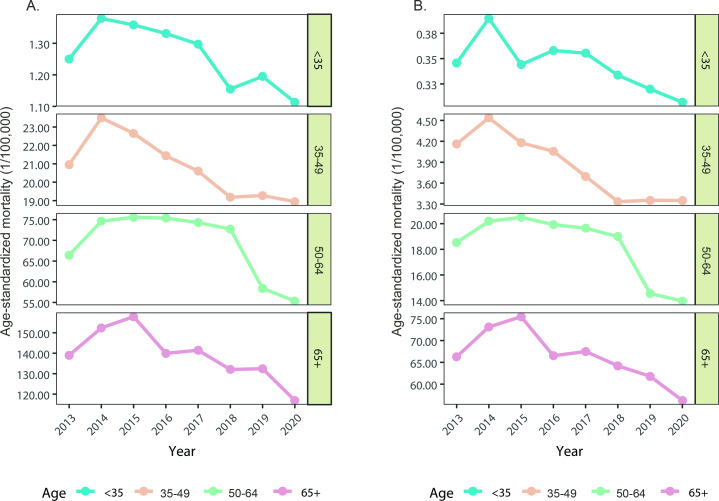
Age-specific liver cancer mortality rat from 2013 to 2020 among men (**A**) and women (**B**) in China.

Liver cancer mortality in all age groups has shown an overall downward trend from 2013 to 2020, but the changes in different age groups still differ (Figure 3). For subpopulations aged<35 and 30-49 years, the mortality increased by 2014 and then persisting declined both in men and women. For those aged 50-64 years, liver cancer mortality was relatively stable before 2018, and a clear downward trend only emerged after 2018. For people aged 65 years and older, the turning point was in 2015 (160.56 per 100,000 in men and 77.01 per 100,000 in women), then decreased to 118.50 per 100,000 in men and 57.17 per 100,000 in women in 2020, respectively.

Multimedia Appendix 4 displayed the spatial distribution of age-specific liver cancer mortality in men from 2013 to 2020. Similar to the distribution of average LASMRs, most regions with high mortality were in the south, especially in Guangxi. The distribution range of areas with high mortality was significantly wider in the 50- to 64-year age group. Apart from the south, high mortality areas were also prevalent in the southwest, east, and northeast. Among women, Guangxi was indeed a high mortality of liver cancer area in the age groups of <35, 65‐49, and 50‐64 years. However, for the age group 65 years and older, the high-mortality area is predominantly concentrated in the northeast, with Heilongjiang, in particular, experiencing mortality ranging from 83.84 to 154.19 per 100,000 in most regions (Multimedia Appendix 5).

### Climate Zones

**Figure 4.**
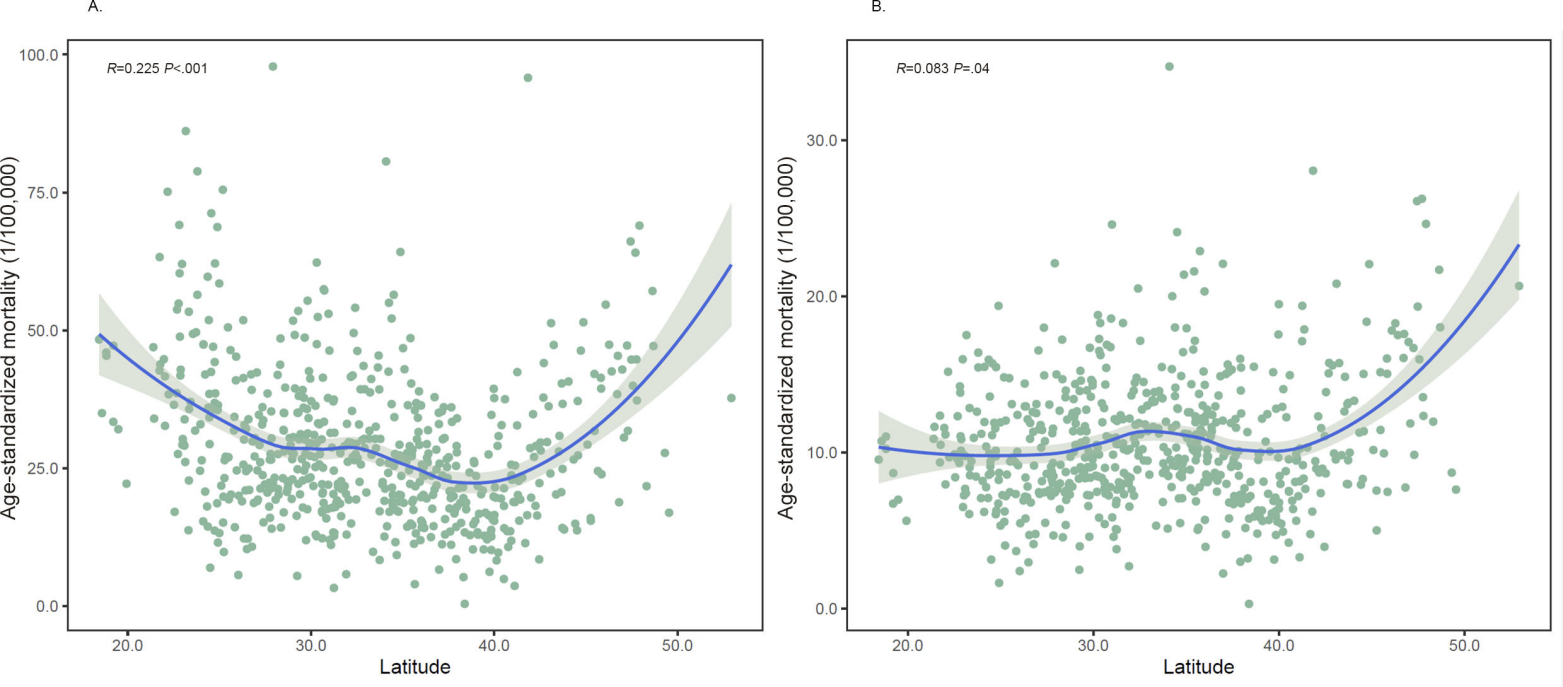
Age-standardized liver cancer mortality rate associated with latitude in men (A) and women (B) in China, 2013-2020.

Partial correlation analysis indicated that the LASMR was related to latitude in China from 2013 to 2020 (Figure 4). However, the associations were opposite between men and women. Among men, the LASMR increased with the decreasing latitude (*R*=0.225; *P*<.001), while LASMR was positively associated with latitude in women (*R*=0.083; *P*=.04). But overall, regardless of gender, there was a positive correlation trend beyond the 40th parallel north, while there was a significant negative correlation trend for men before the 40th parallel north. The trend of LASMRs in different climate classifications from 2013 to 2020 showed specific patterns (Multimedia Appendix 6). In all climate classifications in China, 4 demonstrated notable decreasing trends among men (Cfa, Cwa, Dwa, and Dwb) and 3 among women (Cfa, Cwa, and Dwa). By contrast, except for 2020, the LASMR in Am and Aw showed an upward trend among men but not women. As Multimedia Appendix 7 illustrates, no significant seasonality of LASMRs existed in different climate classifications. Still, among most climate classifications in both men and women, the LASMRs were highest in January and March but lowest in December.

### Years of Life Lost

Multimedia Appendix 8 and Table 3 revealed YLLs and age-standardized YLL rates of liver cancer in China from 2013 to 2020, respectively. Nationwide, the YLLs of liver cancer in China showed an upward trend from 2013 to 2015 and then a gradual decline. Among all years, 2015 had the highest YLL of 16,259,000, and 2014 had the highest YLL rate of 456.46 per 100,000. The YLLs and YLL rates of men were much higher than that of women from 2013 to 2020. Among all age groups, the population aged 50-65 years had the highest YLL, with 6,380,000 in 2020. The YLL rates increased with age, and the YLL rate in people aged 65 years and older was 5698.85 per 100,000 in 2013 and 4850.50 per 100,000 in 2020. Meanwhile, the total YLL of people aged 65 years and older demonstrated a consistent upward trajectory over the years, with a relatively stable YLL rate, except for 2020.

**Table 3.** Age-standardized years of life lost rate (per 100,000) of liver cancer in China, 2013-2020. The climate classification is divided into 11 types based on the Köppen-Geiger classification: Am: tropical monsoon; Aw: tropical savannah; BWk: cold desert; BSk: cold semiarid; Cwa: humid subtropical with dry winter; Cwb: subtropical highland; Cfa: humid subtropical with no dry season; Dwa: humid continental with hot summer; Dwb: humid continental with warm summer; Dwc: humid continental with cold summer; and ET: polar tundra.

	2013	2014	2015	2016	2017
China	410.16	456.46	455.68	435.75	427.32
**Gender**					
Men	571.11	638.13	637.92	612.49	600.78
Women	202.59	222.67	222.62	209.44	205.58
**Age (years)**					
<35	290.93	322.18	312.92	314.68	308.54
35‐49	1401.89	1564.12	1490.95	1415.06	1343.73
50‐64	3122.20	3485.40	3531.87	3526.34	3472.05
65+	5697.85	6266.26	6482.30	5725.15	5804.11
**Region**					
North	230.18	266.81	258.22	260.21	254.45
Northeast	494.92	532.78	522.02	519.04	486.81
East	453.13	461.47	466.07	433.57	426.22
Central	384.02	469.45	471.03	441.76	443.24
South	540.89	647.37	676.21	666.15	658.70
Southwest	439.81	484.01	474.10	451.84	438.29
Northwest	224.08	263.30	292.74	285.65	294.60
**Climate classification**					
Am	399.94	473.00	433.17	548.89	630.49
Aw	461.33	523.32	504.91	637.04	626.50
BWk	230.84	270.57	294.85	286.81	280.48
BSk	176.02	265.33	271.93	272.20	277.89
Cwa	498.73	593.59	577.36	553.40	547.97
Cwb	228.20	289.86	343.30	358.09	321.10
Cfa	439.22	448.78	455.00	428.60	416.66
Dwa	391.46	432.98	431.09	411.81	405.64
Dwb	389.54	440.96	423.50	414.48	400.45
Dwc	535.00	461.17	390.62	355.27	364.77
ET	427.20	408.59	423.75	392.57	414.45

In terms of regional distribution, the total YLLs in the east is the highest, with 4,224,000 in 2020, but the YLL rate in the south is the highest (495.50 per 100,000). Among all regions, the YLL rates in the northeast, central, south, and southwest were higher than those at the national level. Differences in disease burden were also observed in different climate classifications. Cfa had the highest YLL for liver cancer, followed by Cwa and Dwa. Overall, during the period from 2013 to 2020, Cwa consistently exhibited a higher YLL rate. Its peak was in 2014, with a YLL rate of 593.59 per 100,000. Although it decreased to 440.17 per 100,000 by 2020, it remained higher than the national YLL rate.

## Discussion

### Principal Findings

Nationwide, there were 447,392 and 160,490 liver cancer deaths in men and women from 2013 to 2020, respectively. The older population has a higher LASMR and a heavier burden, with the highest YLL rate among people aged 65 years and older. During this period, LASMRs and YLL rates overall decreased, but the total YLLs remained high. The national downward trend was not applied to all regions in China. For instance, an increase could be detected in Guizhou and Chongqing. Moreover, liver cancer mortality demonstrated a variation between gender, age, and region. Most high LASMR regions are concentrated in the south among men and the northeast among women, which is consistent with the relationship between latitude and LASMRs. Spatial dispersion was also embodied in YLL among climate classifications, with Cwa having the highest YLL rate.

The downward trend in the past 8 years could be mainly attributed to the control of hepatitis B. Starting in 1992, the hepatitis B vaccine was introduced for the first time into the scheduled immunization program for newborns and infants in China, leading to an annual increase in vaccine coverage rates [23]. By 2002, China had the vaccine into a national expanded program on immunization, providing free vaccinations for newborns and infants, and as of 2015, the hepatitis B vaccine coverage rate of newborns has reached 99.6% [23]. Our research findings suggested that this initiative has yielded substantial benefits in recent years. In 2020, the average LASMR in China was 26.38 among men and 9.83 among women per 100,000, compared to 28.79 and 11.09 per 100,000 in 2013, indicating a slight decrease. It is worth noting that the potential behavior change caused by the pandemic in 2020 may have led to some confusion in reporting non-COVID-19 cases [24], resulting in an underestimate of liver cancer mortality. Nevertheless, these figures still exceed the global average reported by GLOBOCAN 2020, which is 12.9 in men and 4.8 people in women per 100,000 worldwide [25]. This implies that despite the encouraging downward trend shown over the period, the liver cancer situation in China remains notably high.

Significant spatial variations of LASMR were observed in this study, which is similar to a recent paper exploring liver cancer incidence in China. However, a study found that the southwest had the highest liver cancer incidence, whereas our study showed higher rates in the south may be due to the bias of that study’s data being based on only 5 cancer registries [26]. Guangxi had the highest LASMR in men and Heilongjiang in women. Additionally, there were regional differences in LASMR between men and women, with higher rates in the southern part for men (including Guangxi, Guangdong, and Hainan) and higher rates in the northeast for women (including Heilongjiang and Jilin). Notably, the differences among men seem more pronounced, with Guangxi having the highest LASMR being approximately 3.8 times higher than Xinjiang, which had the lowest LASMR, while among women, this difference was about 2.5 times higher. Regions with high LASMRs, both in men and women, did not align with the geographic distribution of hepatitis B incidence, which is a primary driver of liver cancer. Between 2005 and 2010, the highest incidence of hepatitis B in China was mainly in the northwest region [27], manifesting that the distribution of liver cancer mortality in China between 2013 and 2020 may be influenced by factors other than hepatitis B.

The high prevalence of alcohol use among women in the northeast may partially explain the high LASMR detected in areas such as Heilongjiang and Jilin [28]. However, among men, regions with high LASMR do not always have a high prevalence of alcohol use [28], like Guangxi.

From an environmental perspective, we have discovered some intriguing associations. Most areas with high LASMR are situated in the Cwa and Cfa climate (Multimedia Appendix 9), which are humid subtropical regions (Multimedia Appendix 1). This correlation could potentially link liver cancer to a climate-related carcinogen, aflatoxin. Aflatoxin is produced by *Aspergillus flavus* and *Aspergillus parasiticus* [29]. *Aspergillus flavus* commonly contaminates grains and nuts and significantly impacts every stage of their journey from planting, processing, and storage to eventual consumption by humans [29]. The growth of *A. flavus* is affected by temperature, with its optimal growth occurring in the temperature range between 28 and 37 °C, while subtropical regions are precisely in this favorable temperature range [30].

A study by Chen et al [31] revealed that in China, Guangxi and Guangdong, population attributable fraction of aflatoxin exposure was found to be 4.48% and 2.25%, respectively, making them the 2 provinces with the highest levels of aflatoxin exposure in China. Interestingly, in our study, the south, which encompasses these 2 provinces, exhibited the highest YLL rate. Furthermore, climate classifications of these 2 regions characterized by the Cwa and Cfa also demonstrated relatively high YLL rates. Another suspicious risk factor to consider is liver fluke infection, which has been identified as a significant risk factor for intrahepatic cholangiocarcinoma, a subtype of liver cancer, with an odds ratio of 4.69 for the risk of liver cancer [32]. Guangxi and Guangdong are the most prevalent areas of liver flukes in China, mainly due to dietary habits that include the consumption of raw fish [33]. Similar to *A. flavus*, liver fluke infections are more likely to occur in warm environments [34].

The overall decline in liver cancer in China is gratifying, but we also found in our study that regional heterogeneity still exists, as well as inconsistent burdens between populations. Therefore, it seems not a wise choice to stand on the sidelines immediately after enjoying the dividends brought by hepatitis virus control. Changes in risk factors in different regions may alter the optimistic trend and distribution of liver cancer in China in the future. It is predicted that the liver cancer incidence in the south of China will increase by 86.9% in 2032 compared to 2012 [26]. Moreover, taking the development of liver cancer in high-income countries as a warning, such concerns are reasonable. Although the alarming growth trends in these countries cannot be simply attributed to a specific factor, environmental changes may potentially account for it. The Intergovernmental Panel on Climate Change’s [35] AR6 Synthesis Report suggested that the goal of keeping global warming below 1.5 °C in the 21st century will be difficult to achieve, but that an increase of 2 °C is within reach. The United Nations has also declared that the era of global boiling has arrived [36].

The impact of climate change on liver cancer is likely to be multifaceted. Some studies have indicated that aflatoxin will inevitably be affected by climate change, and the prevalence of aflatoxin will expand under climate change [37,38]. Due to climate change, the effects of aflatoxin and other mycotoxins on crops have escalated, impacting 60% to 80% of yields [39]. Globally, the high incidence of liver cancer is predominantly located in countries with hot and humid climates and lacking aflatoxin management [40]. In such climates, *A. flavus* is more likely to breed [40]. Moreover, the distribution of climate zones will shift. Tropical and subtropical regions are expected to expand [41], undoubtedly creating more favorable conditions for the production of aflatoxins. The entire process from the production to the intake of aflatoxin may be facilitated by climate change [29], thereby increasing the risk of liver cancer.

Additionally, the prevalence of liver flukes may also be unable to escape the impact of climate change [42]. Beyond its direct impact on carcinogens, climate change could lead to the disruption of cancer care facilities, affecting the accessibility of patients with cancer to treatment and care, which can have catastrophic consequences for patients with cancer and may increase their risk of death [43]. In our study, we indeed observed distinct climate patterns associated with the distribution of liver cancer mortality. This finding highlights the need to proactively address cancer prevention and control in the face of these environmental changes. Efforts should be particularly focused on regions sensitive to climate change and on vulnerable populations, such as the older population. Additionally, cancer surveillance systems could be integrated with environmental monitoring systems to achieve more precise prevention and control strategies.

### Strengths and Limitations

Our research used the latest and representative data from the NDSS to describe the distribution of liver cancer mortality in all 31 provinces and cities in Mainland China, considering both spatial and temporal dimensions. In addition to age-standardized mortality, we also calculated the YLL and YLL rate to understand the burden of liver cancer in China, encompassing different population groups from 2013 to 2020. Furthermore, to our knowledge, it is the first study to describe the mortality and burden of liver cancer in China through the lens of various climate classifications. This study also has some limitations. First, there is variability in different types of liver cancer, but the distribution of liver cancer subtypes cannot be characterized due to data inaccessibility. Second, due to the lack of national data on risk factors related to liver cancer, our study is unable to compare the distribution pattern of liver cancer in China with the corresponding distribution of risk factors. This limitation prevents us from delving more deeply into the underlying reasons for the disparate distribution of liver cancer.

### Conclusions

The overall LASMR in China from 2013 to 2020 showed a downward trend, but it remains significantly higher than the global average. Meanwhile, there were noticeable variations in the geographical distribution of LASMR within China. High LASMR in men was mainly concentrated in the south, while women were concentrated in the northeast. The climatic distribution of LASMR in men is primarily in subtropical areas, which may relate to aflatoxin and liver fluke. More research on climate and liver cancer carcinogens is urgently required to deeply explore the relationship between climate and environmental factors and the distribution of liver cancer at finer spatial resolution. Furthermore, the burden of liver cancer also varied among different groups, with higher YLL rates in the older population, south, and subtropical regions. Future liver cancer prevention and control strategies should prioritize these high-risk areas and populations to address potential shifts in the distribution and trend of liver cancer in the future.

## Supplementary material

10.2196/54967 Multimedia Appendix 1 Köppen-Geiger climate classification legend.

10.2196/54967 Multimedia Appendix 2 Trend of liver cancer mortality from 2013 to 2020 among men (A) and women (B) in China.

10.2196/54967 Multimedia Appendix 3 Spatial distribution of gender-specific age-standardized liver cancer mortality in China (2013-2016 and 2017-2020).

10.2196/54967 Multimedia Appendix 4 Spatial distribution of age-specific liver cancer mortality among men in China, 2013-2020.

10.2196/54967 Multimedia Appendix 5 Spatial distribution of age-specific liver cancer mortality among women in China, 2013-2020.

10.2196/54967 Multimedia Appendix 6 Age-standardized liver cancer mortality from 2013 to 2020 by climate classification among men (A) and women (B) in China. The climate classification is divided into 11 types based on the Köppen-Geiger classification.

10.2196/54967 Multimedia Appendix 7 Monthly patterns in liver cancer mortality among men (A) and women (B) in China, 2013-2020.

10.2196/54967 Multimedia Appendix 8 Total years of life lost (10,000) of liver cancer in China (2013-2020).

10.2196/54967 Multimedia Appendix 9 Köppen-Geiger climate classification in China.

## Abbreviations

AAPC: average annual percentage change
DALY: disability-adjusted life year
DSP: Death Surveillance Point
EAPC: estimated annual percentage change
*ICD-10*: *International Classification of Diseases, Tenth Revision*
LASMR: age-standardized mortality rate of liver cancer
NDSS: National Death Surveillance System
YLL: years of life lost
