# Supplementary material for: Liver Cancer Mortality Disparities at a Fine Scale Among Subpopulations in China: Nationwide Analysis of Spatial and Temporal Trends
Source: JMIR Public Health Surveill. 2024 Aug 8;10:e54967. doi: 10.2196/54967 (PMC11327839; doi:10.2196/54967)
Supplement: Multimedia Appendix 1 [file publichealth-v10-e54967-s001.docx]

Multimedia Appendix 1. Köppen Geiger climate classification legend

| Climate code | Legend |
| --- | --- |
| Am | Tropical, monsoon |
| Aw | Tropical, savannah |
| BWk | Arid, desert, cold (cold desert) |
| BSk | Arid, steppe, cold (cold semi-arid) |
| Cwa | Temperate, dry winter, hot summer (humid subtropical) |
| Cwb | Temperate, dry winter, warm summer (subtropical highland) |
| Cfa | Temperate, no dry season, hot summer (humid subtropical) |
| Dwa | Cold, dry winter, hot summer (humid continental) |
| Dwb | Cold, dry winter, warm summer (humid continental) |
| Dwc | Cold, dry winter, cold summer (subarctic) |
| ET | Polar, tundra |
