# Supplementary figures and images for: Liver Cancer Mortality Disparities at a Fine Scale Among Subpopulations in China: Nationwide Analysis of Spatial and Temporal Trends
Source: JMIR Public Health Surveill. 2024 Aug 8;10:e54967. doi: 10.2196/54967 (PMC11327839; doi:10.2196/54967)

A. Men

Men: 1 Joinpoint

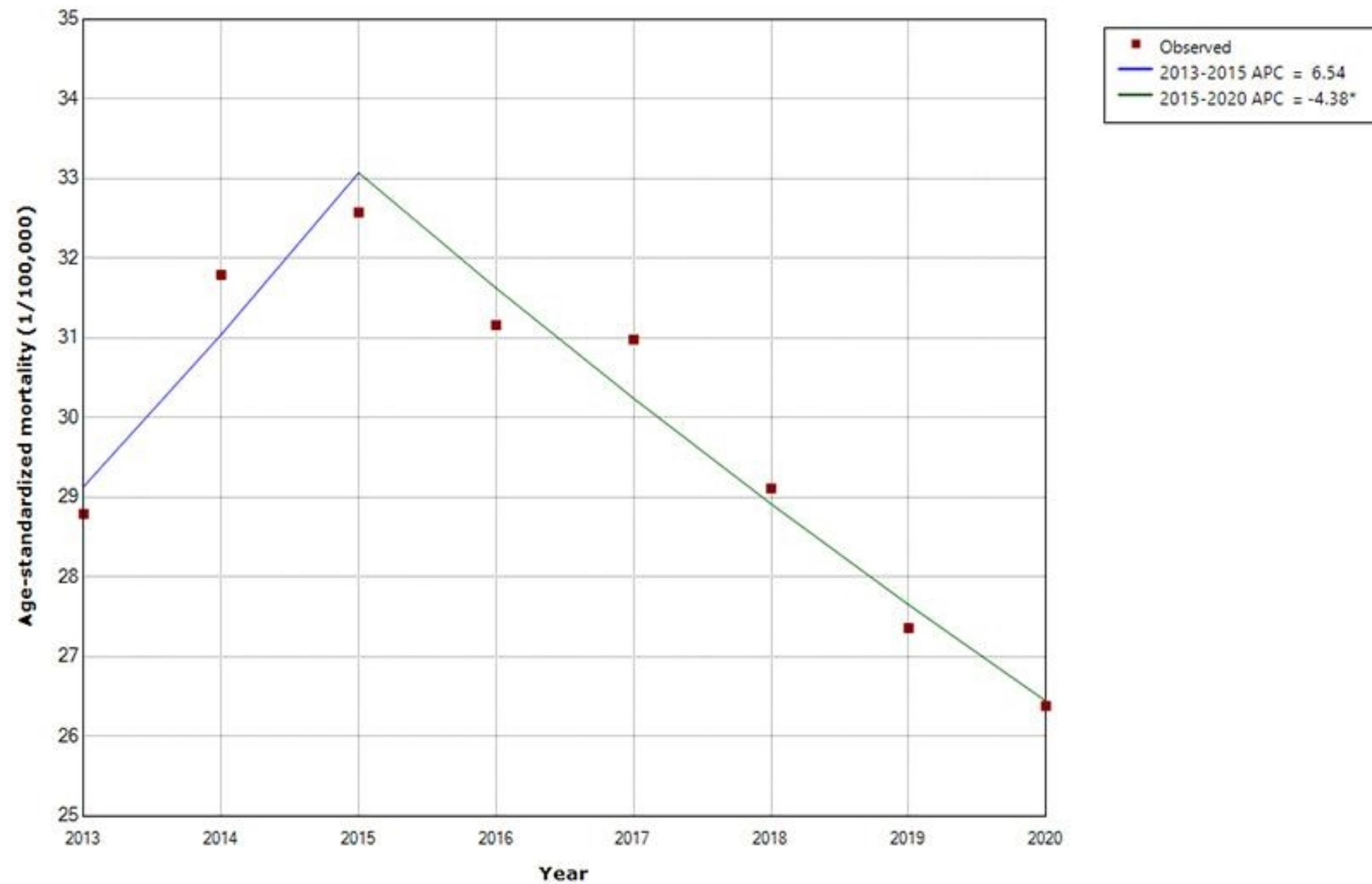

B. Women

Women: 1 Joinpoint

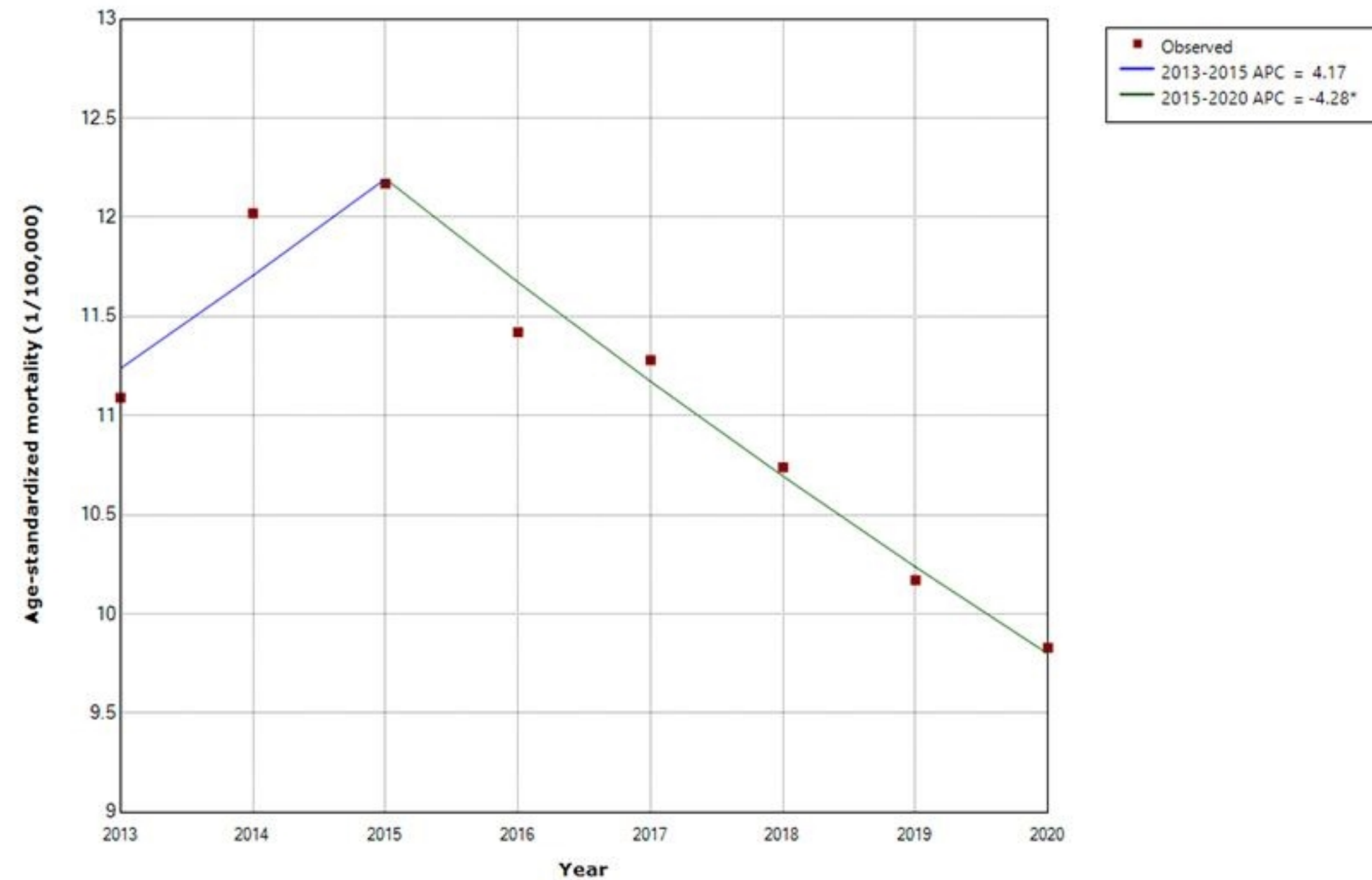

Supplement: Multimedia Appendix 2 [file publichealth-v10-e54967-s002.pdf]

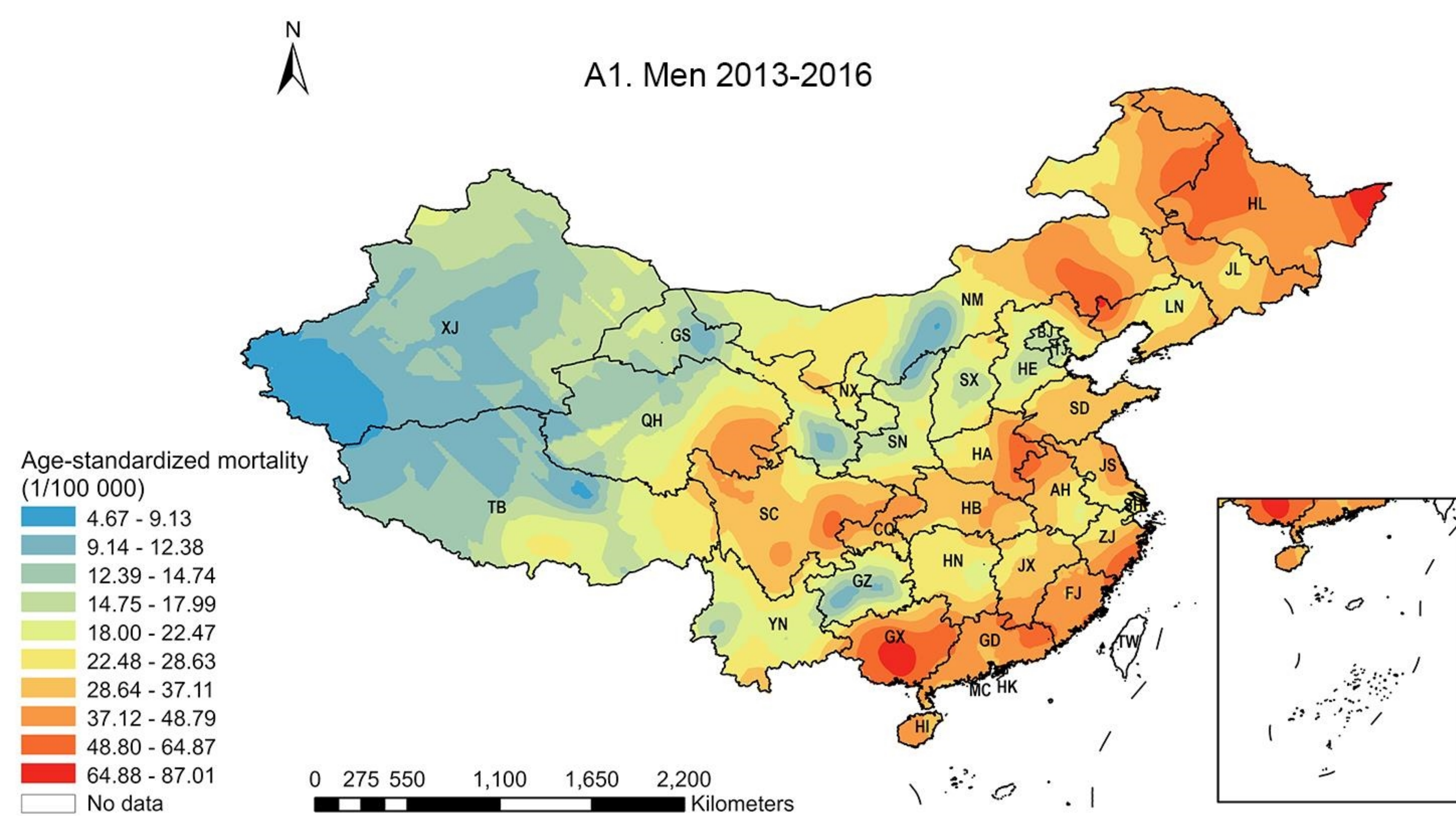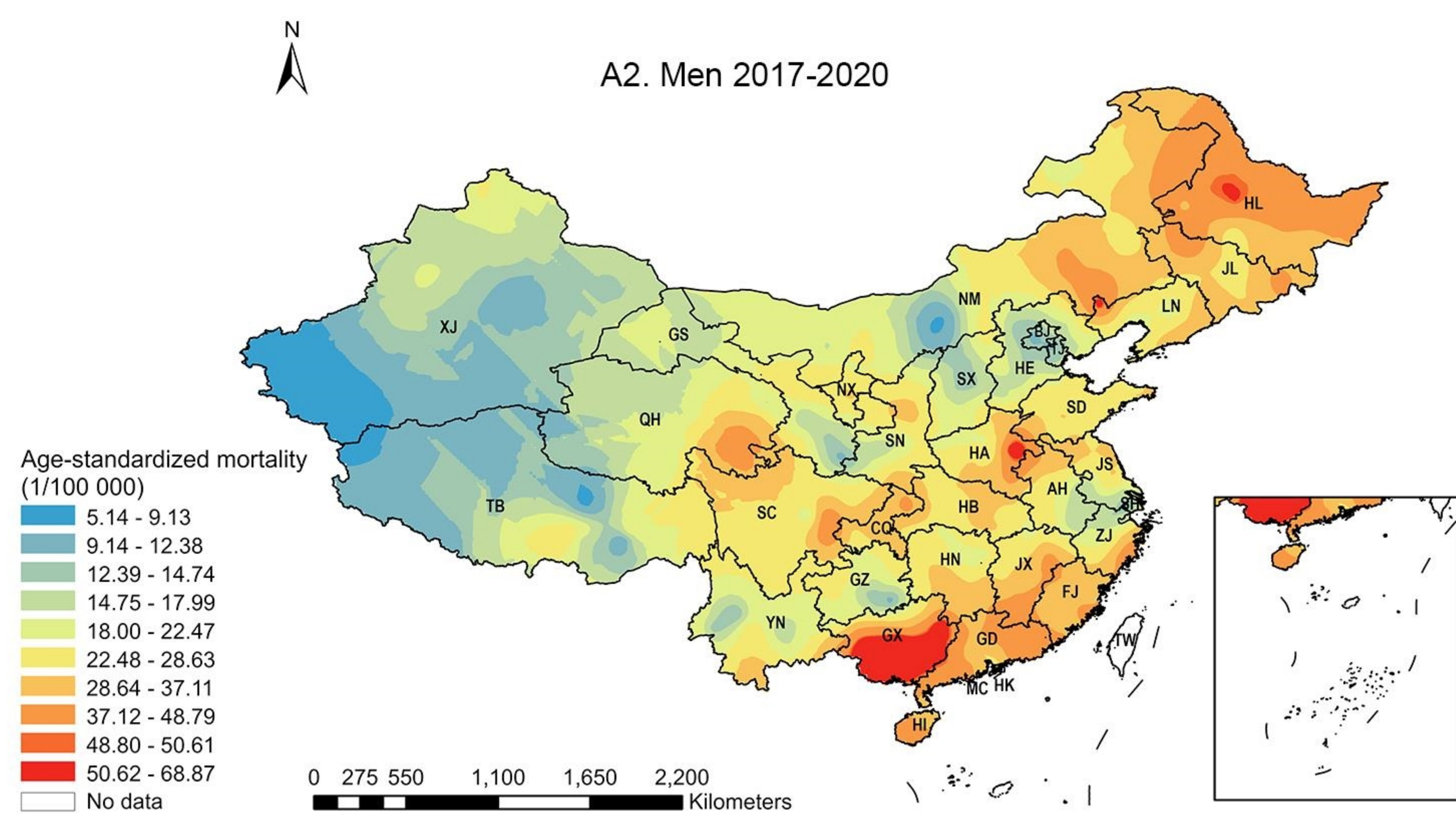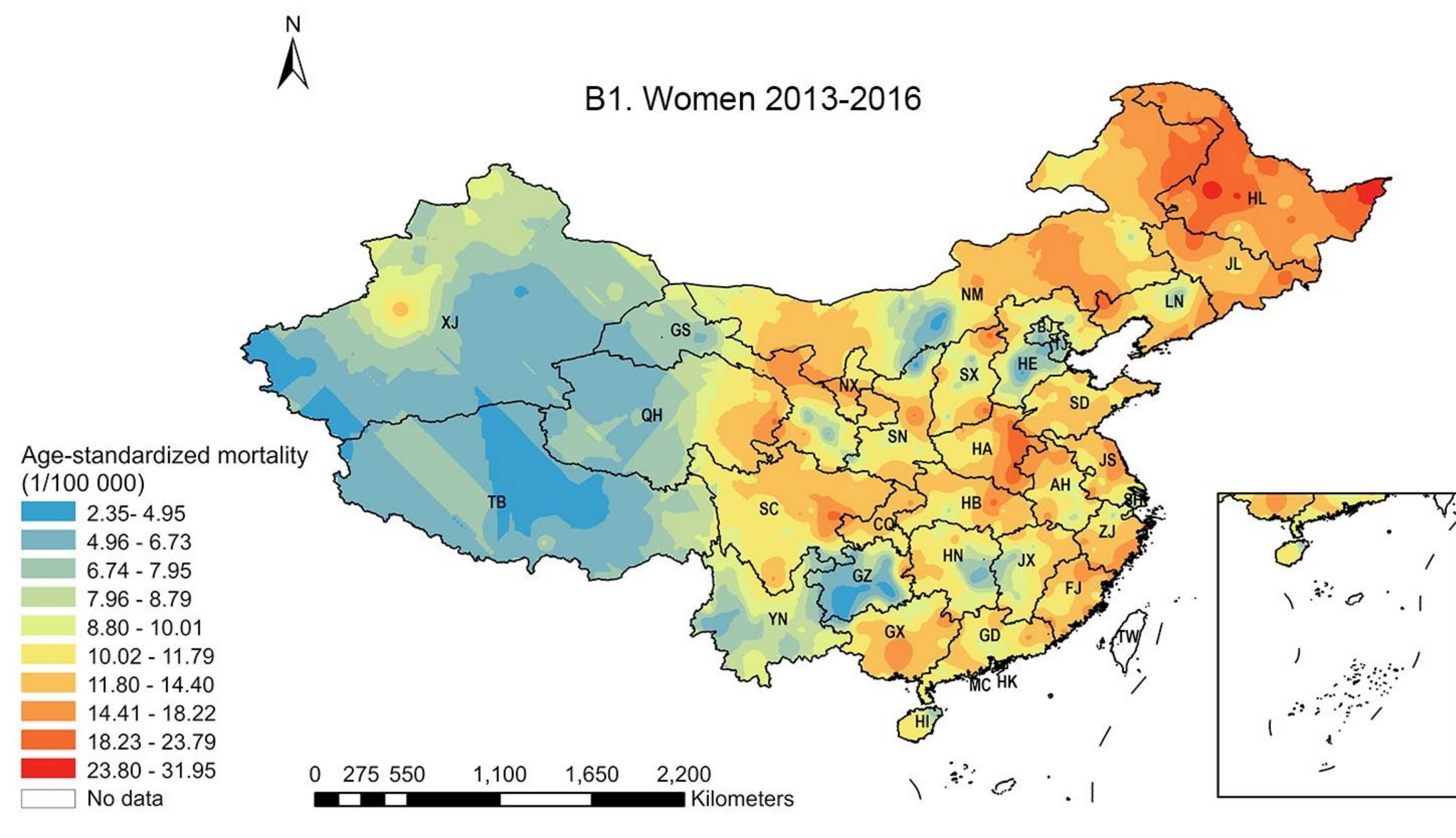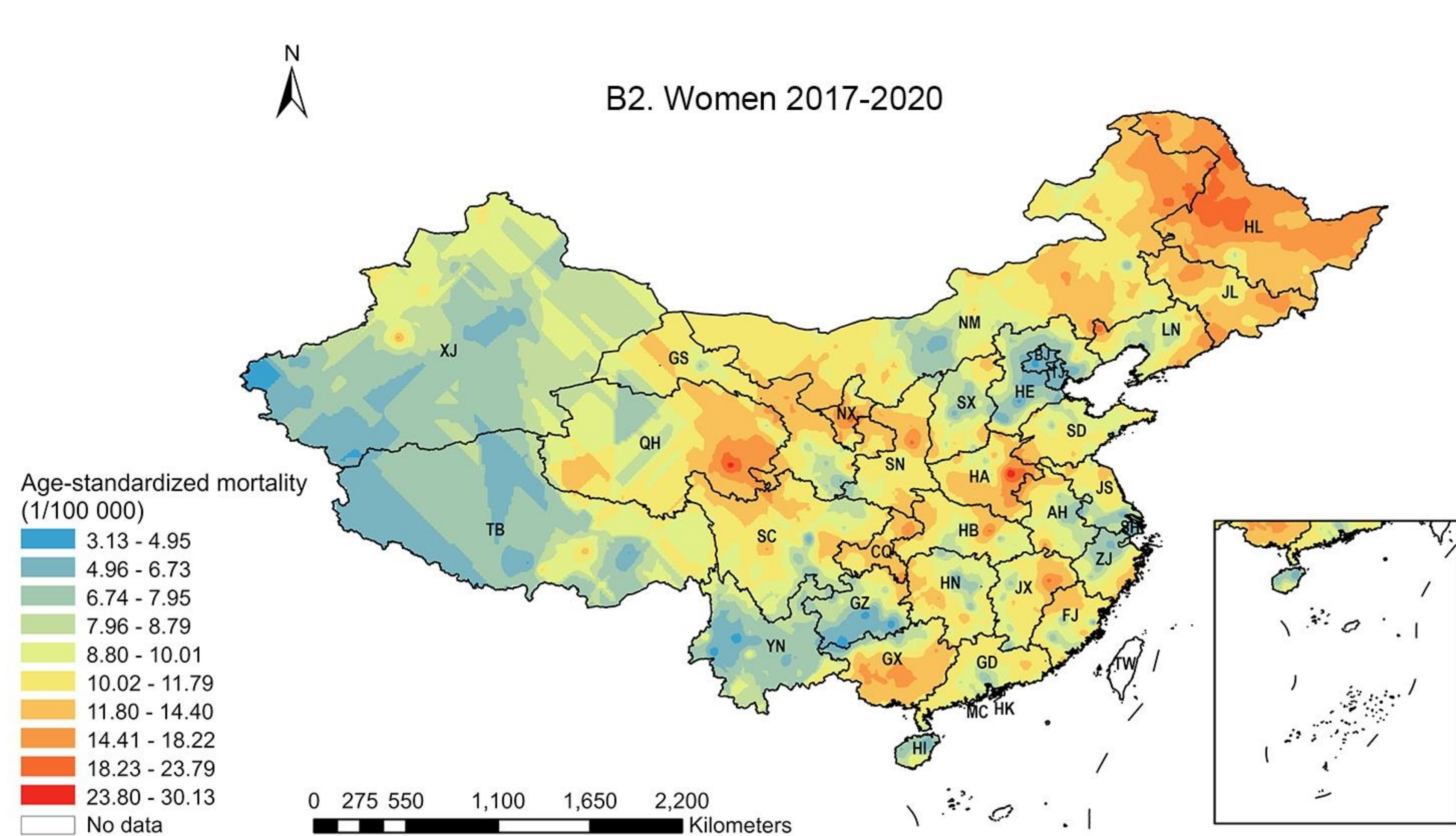

Supplement: Multimedia Appendix 3 [file publichealth-v10-e54967-s003.pdf]

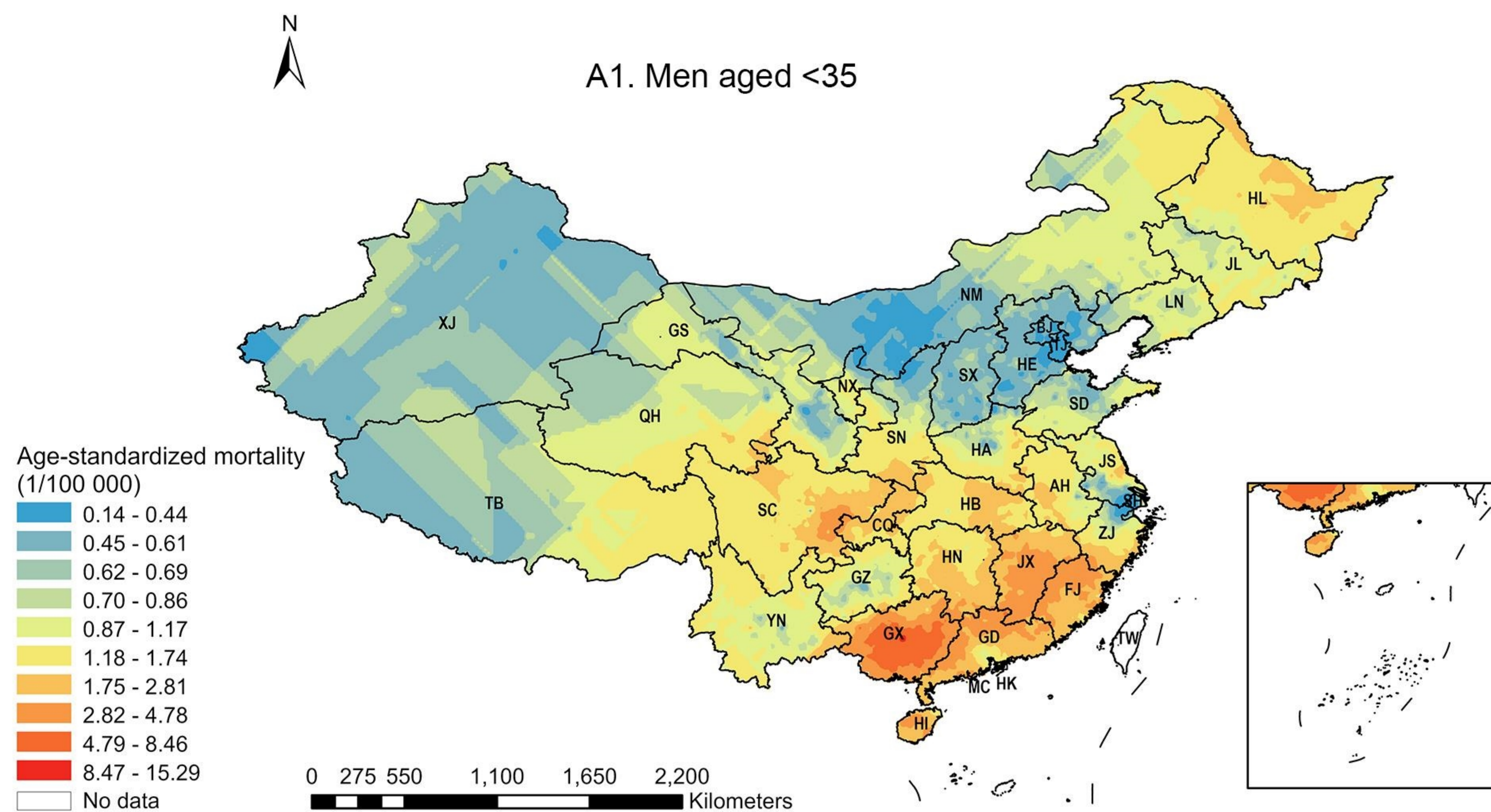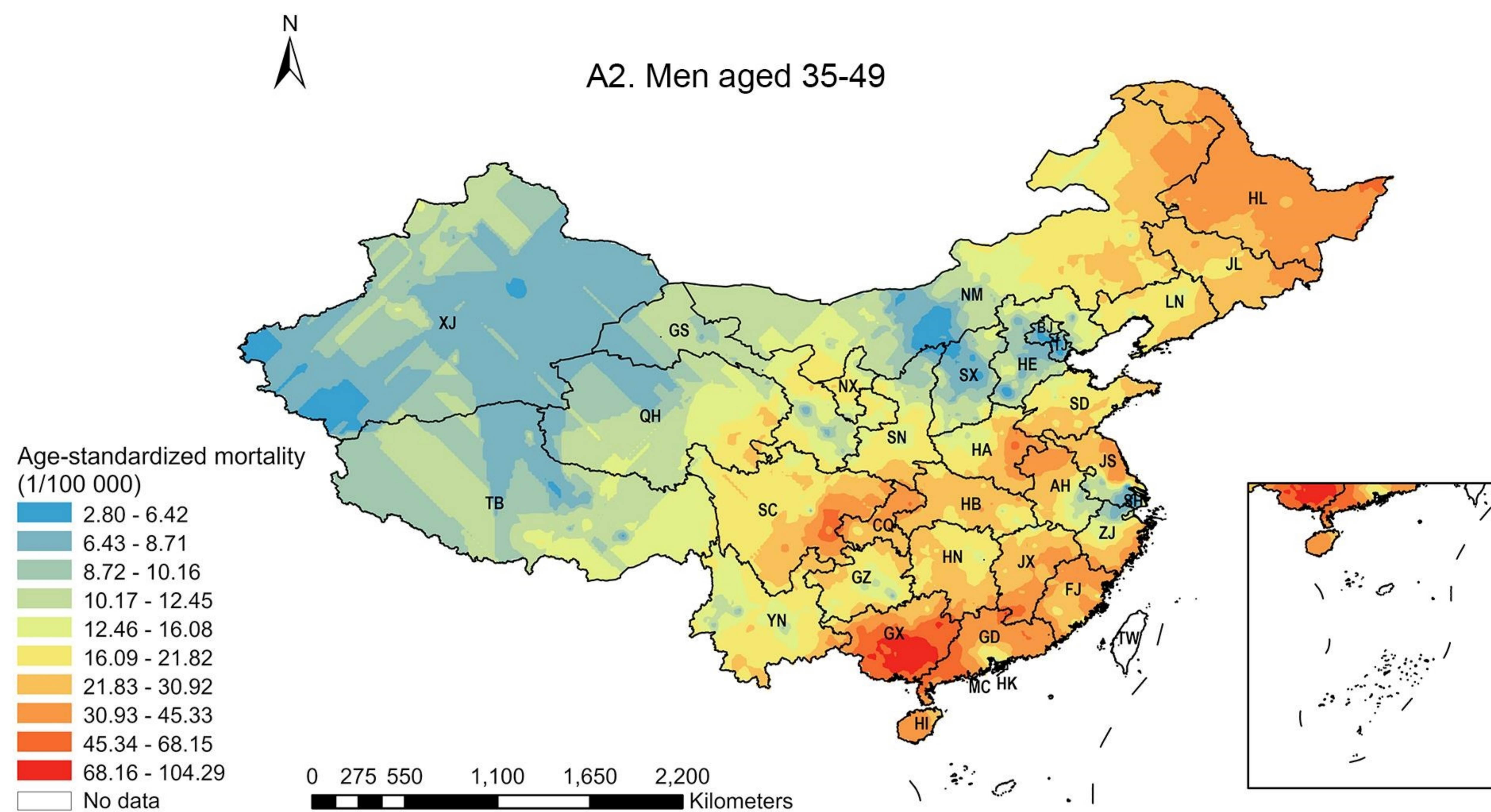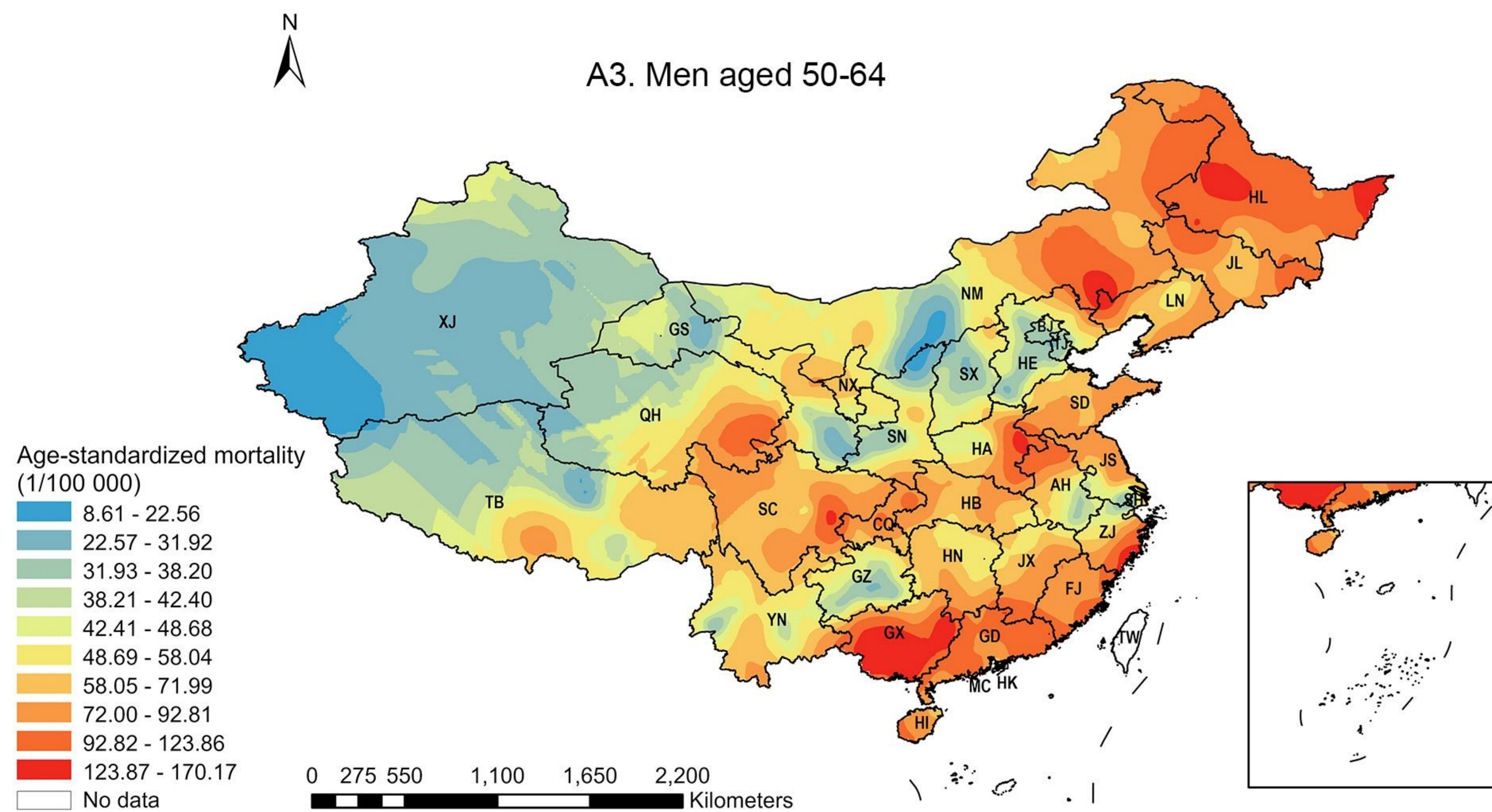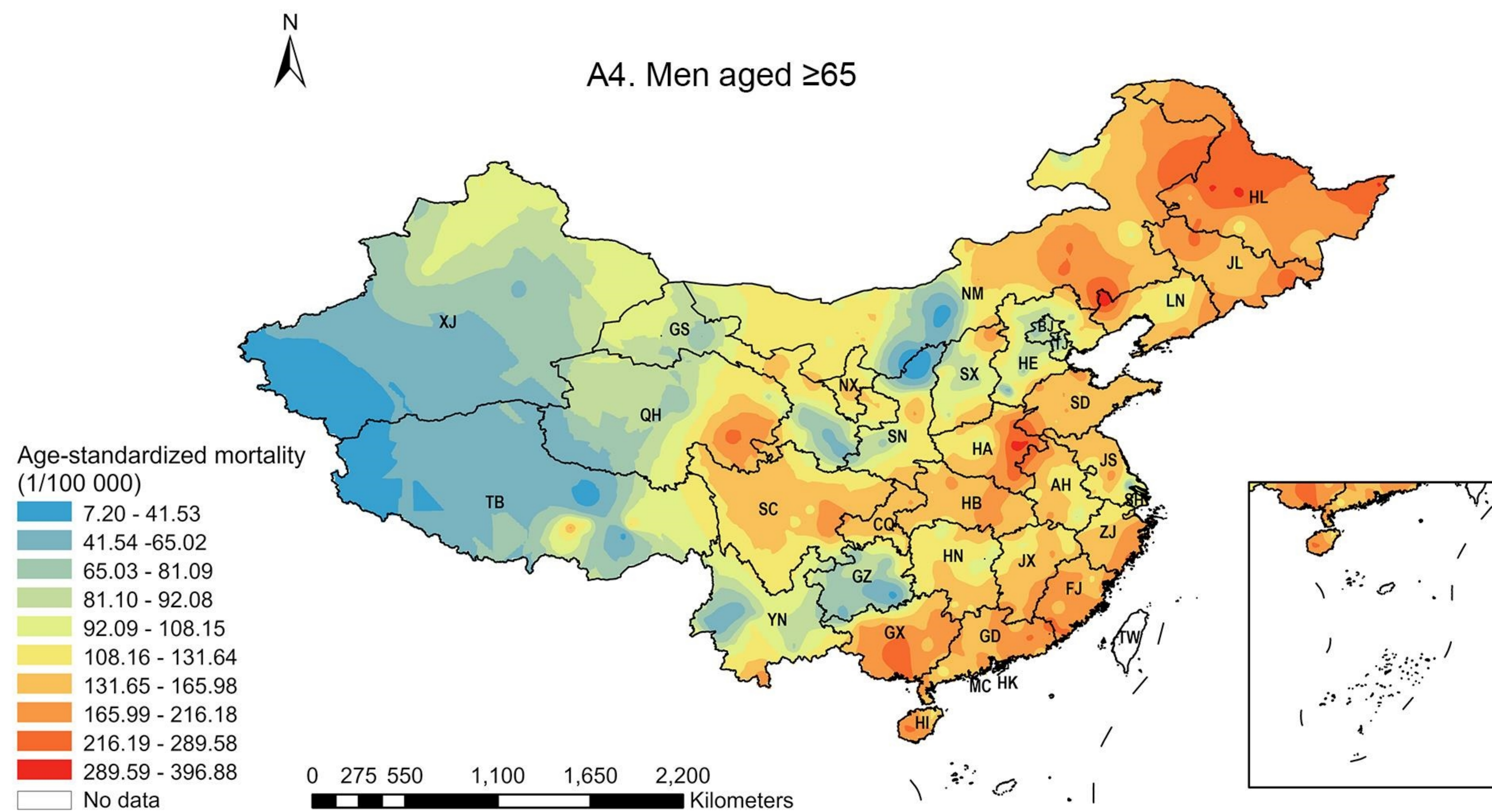

Supplement: Multimedia Appendix 4 [file publichealth-v10-e54967-s004.pdf]

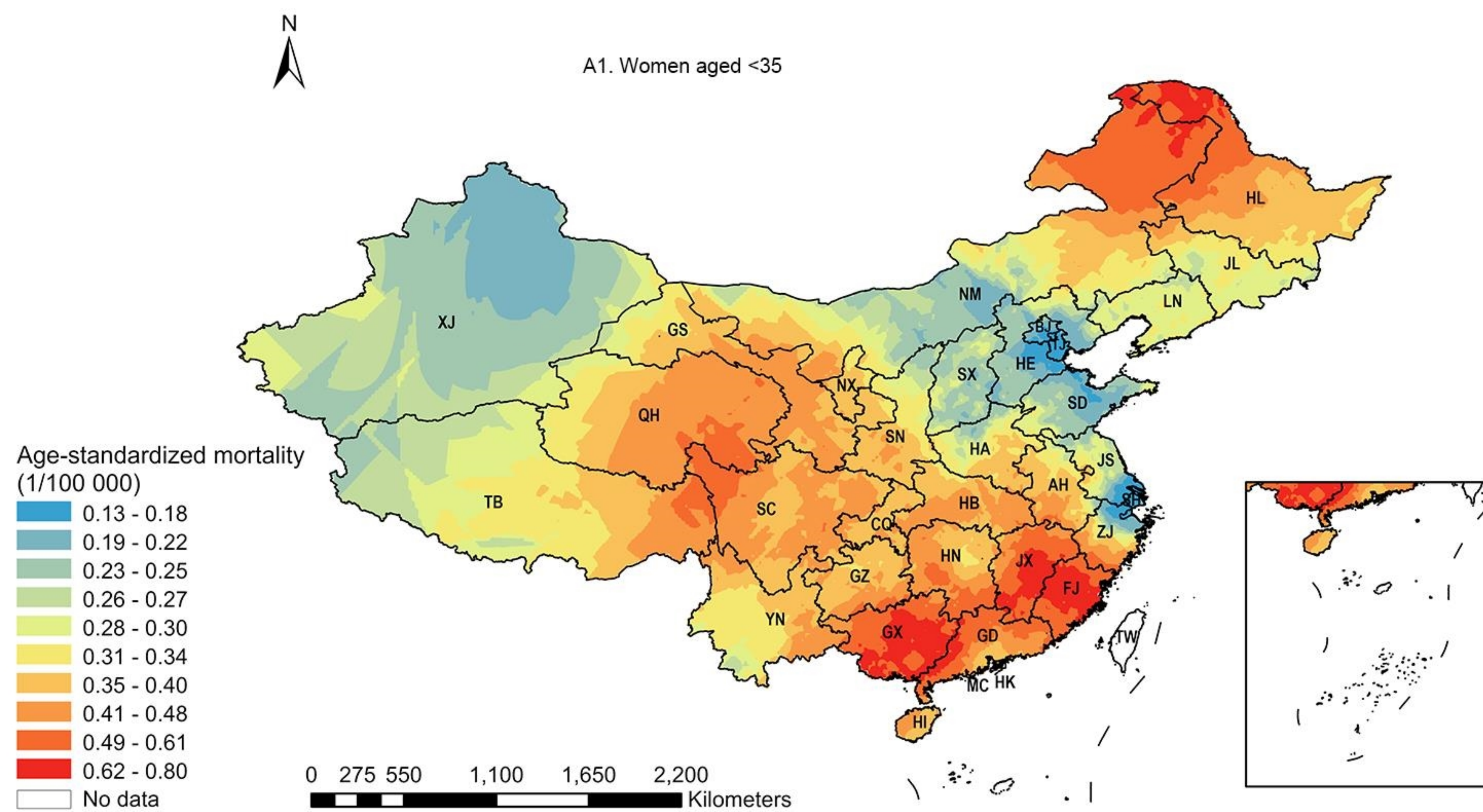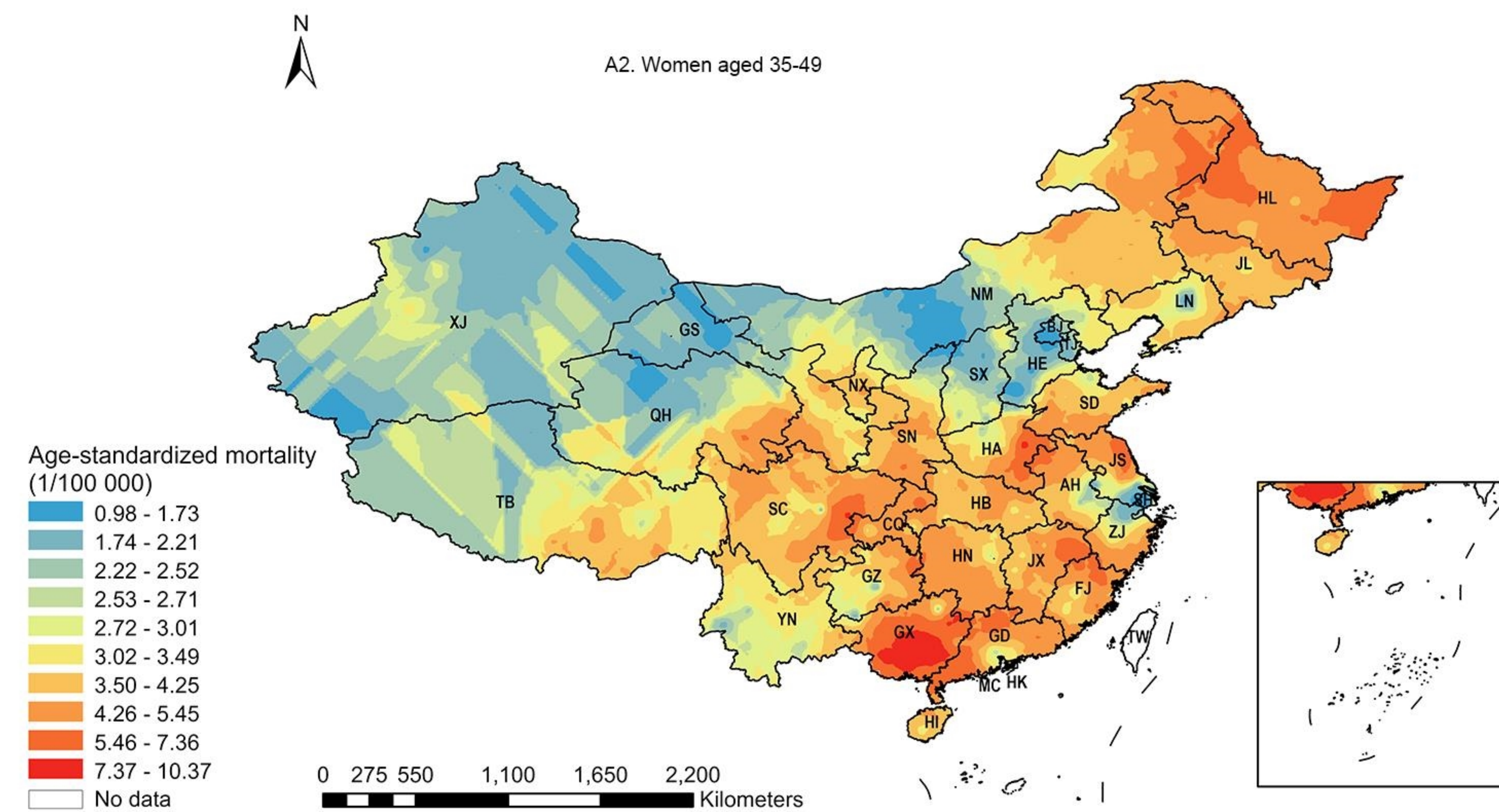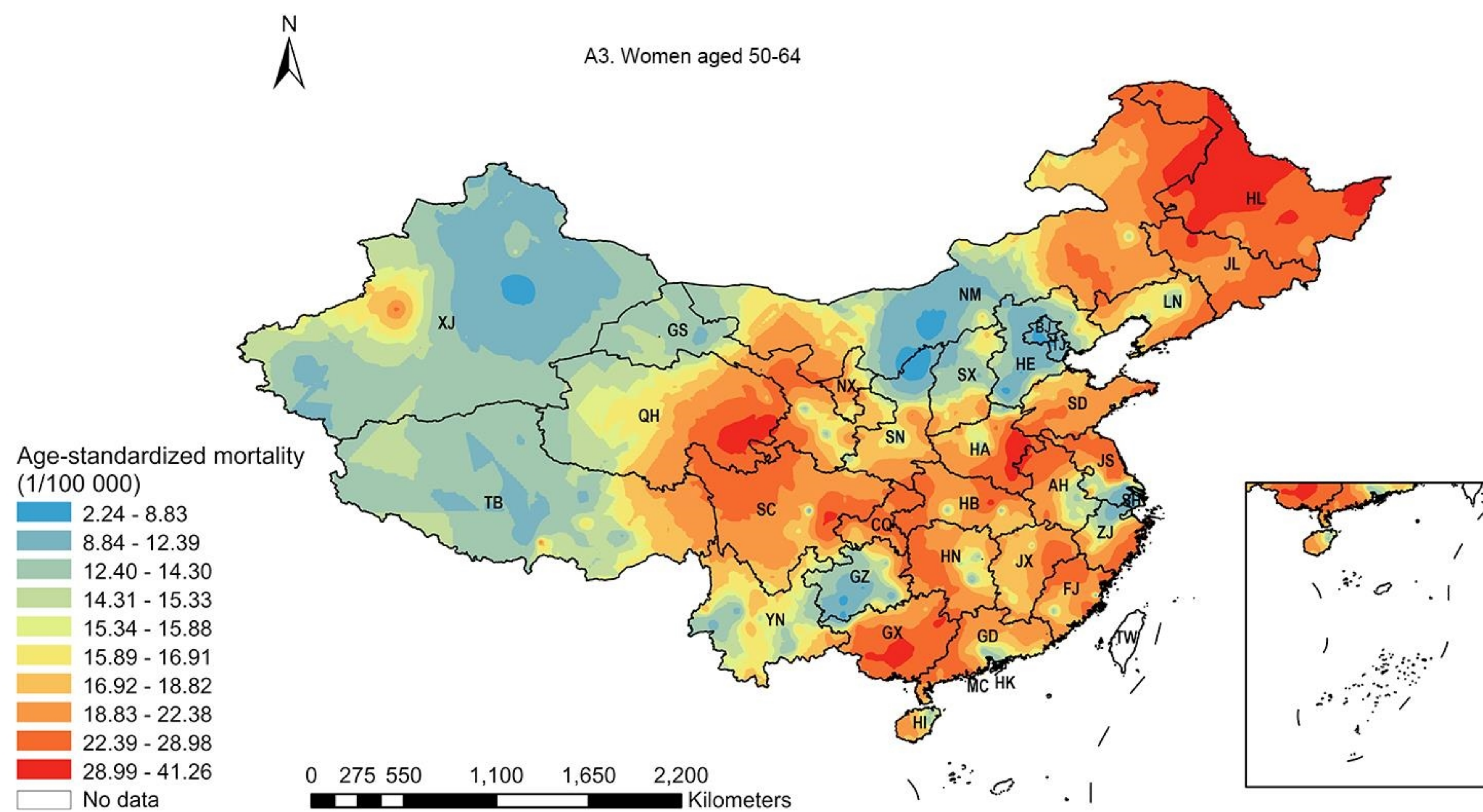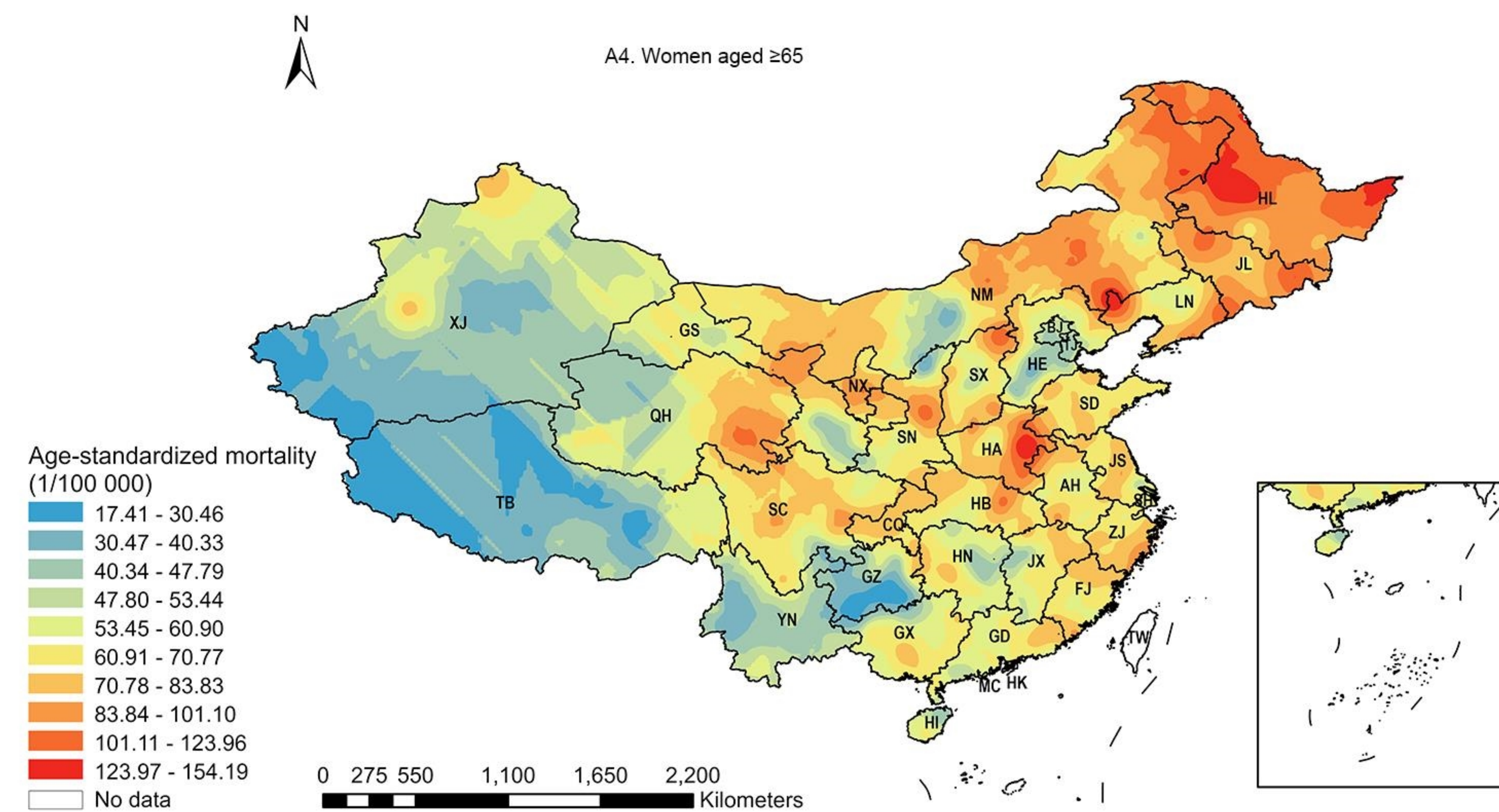

Supplement: Multimedia Appendix 5 [file publichealth-v10-e54967-s005.pdf]

A. Men

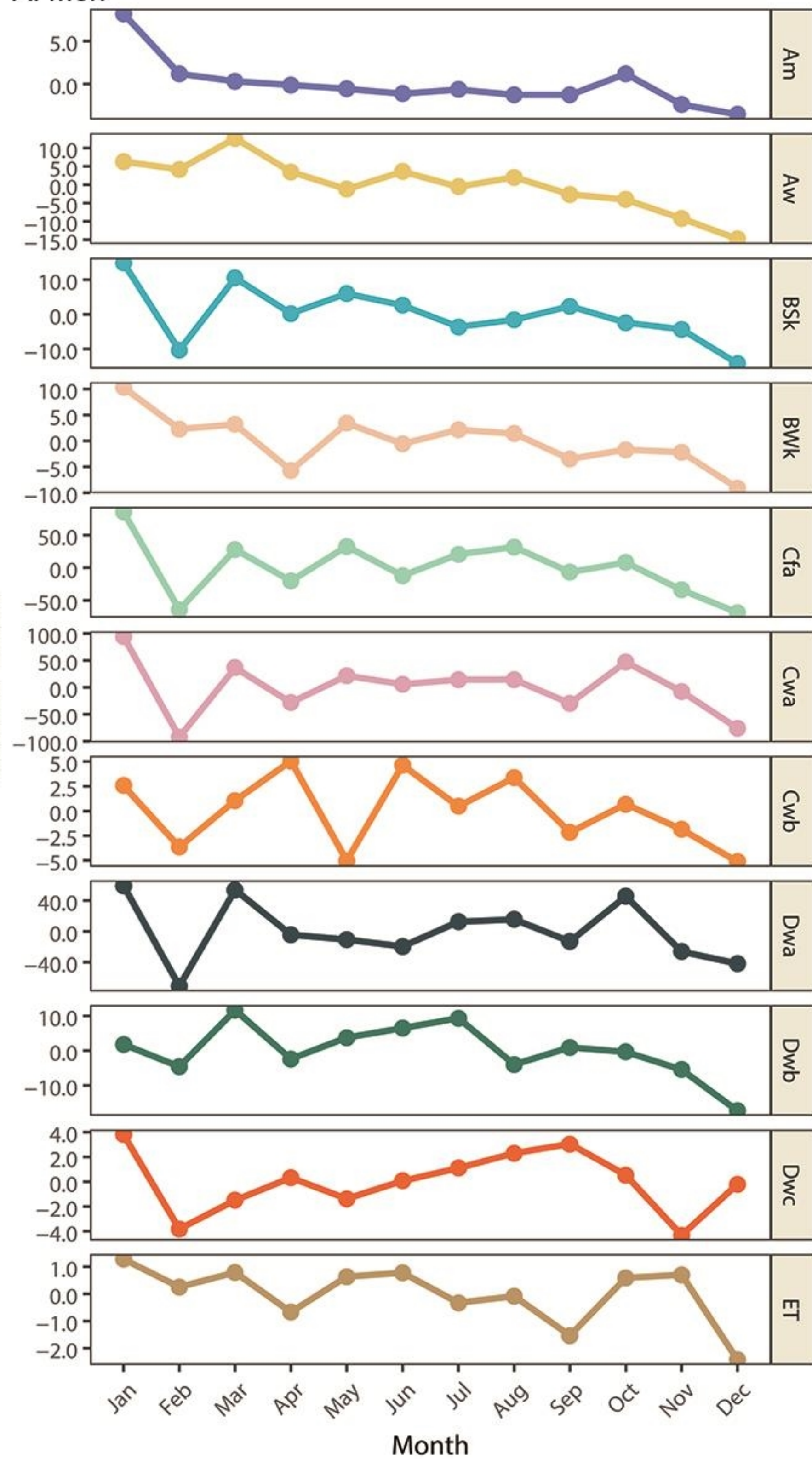

B. Women

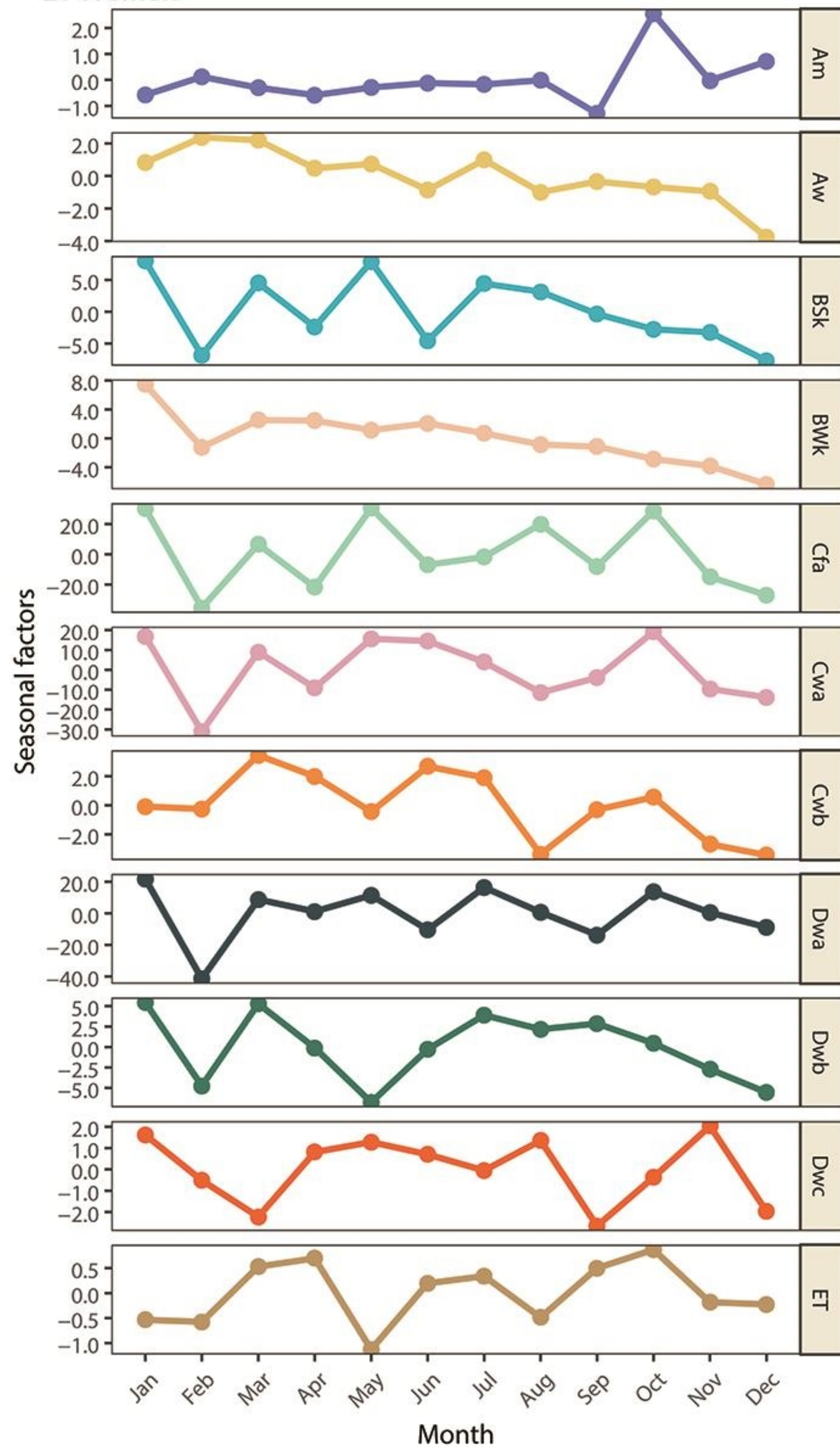

Supplement: Multimedia Appendix 7 [file publichealth-v10-e54967-s007.pdf]

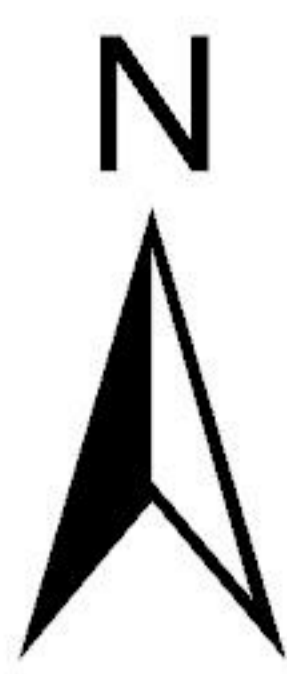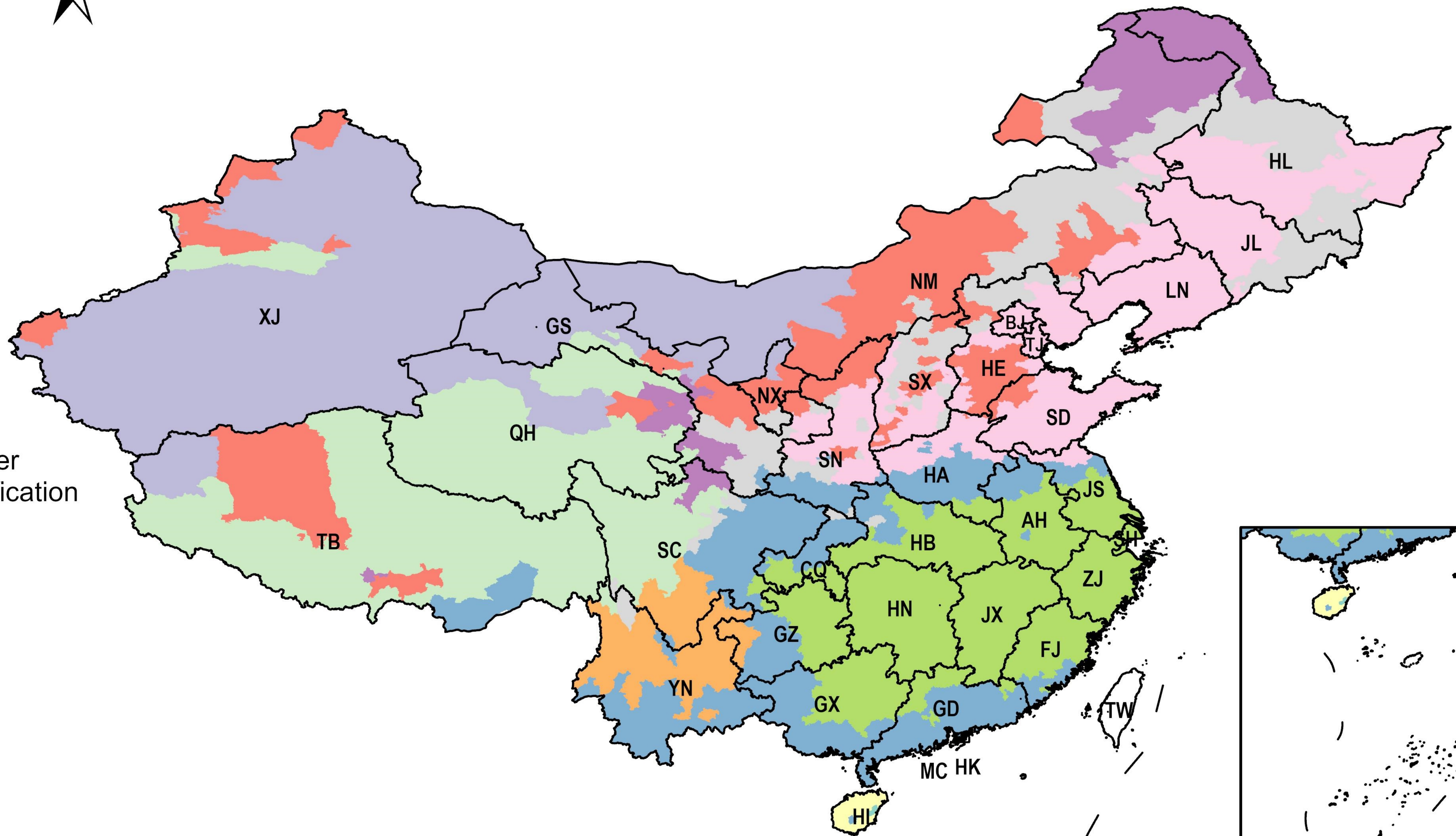

Köppen Geiger  
climate classification

- 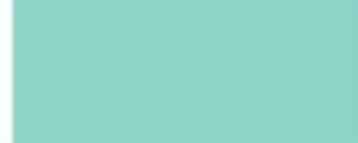 Am
- 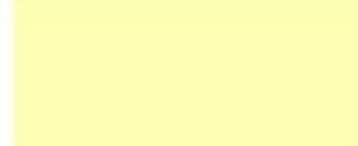 Aw
- 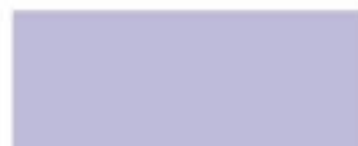 BWk
- 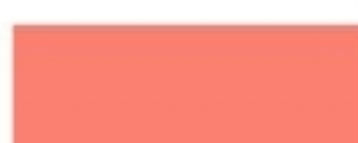 BSk
- 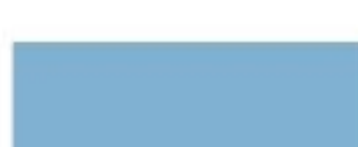 Cwa
- 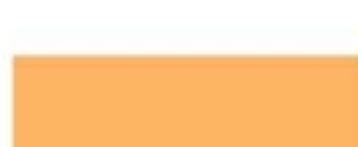 Cwb
- 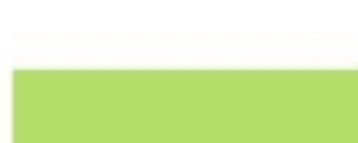 Cfa
- 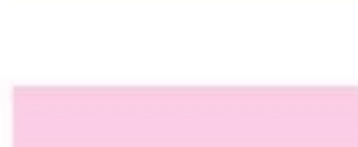 Dwa
- 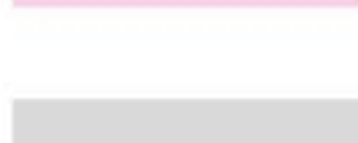 Dwb
- 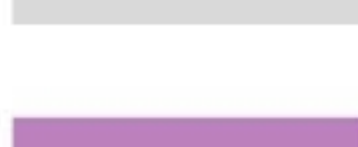 Dwc
- 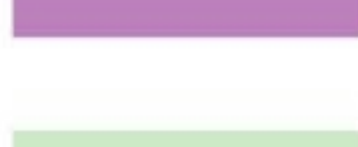 ET
- 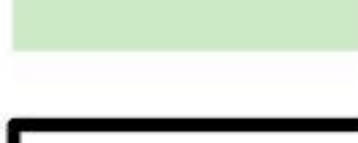 No data

0 275 550 1,100 1,650 2,200  
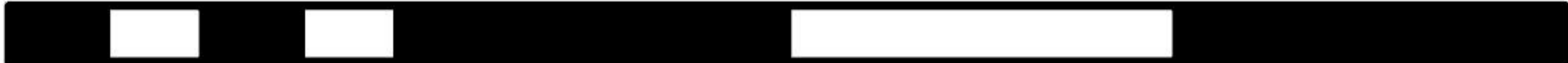 Kilometers

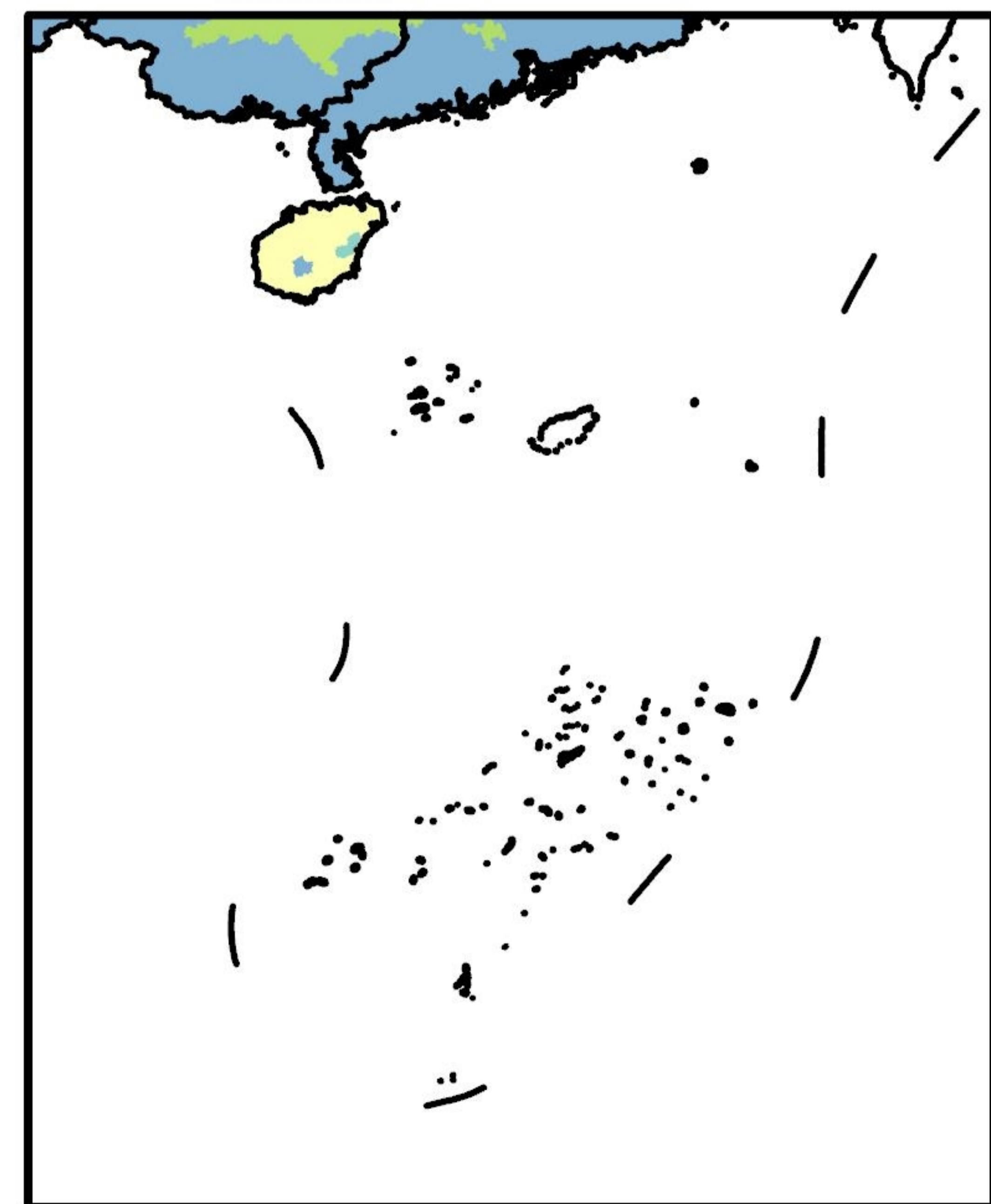

Supplement: Multimedia Appendix 9 [file publichealth-v10-e54967-s009.pdf]
