# Supplementary material for: Liver Cancer Mortality Disparities at a Fine Scale Among Subpopulations in China: Nationwide Analysis of Spatial and Temporal Trends
Source: JMIR Public Health Surveill. 2024 Aug 8;10:e54967. doi: 10.2196/54967 (PMC11327839; doi:10.2196/54967)
Supplement: Multimedia Appendix 6 [file publichealth-v10-e54967-s006.pdf]

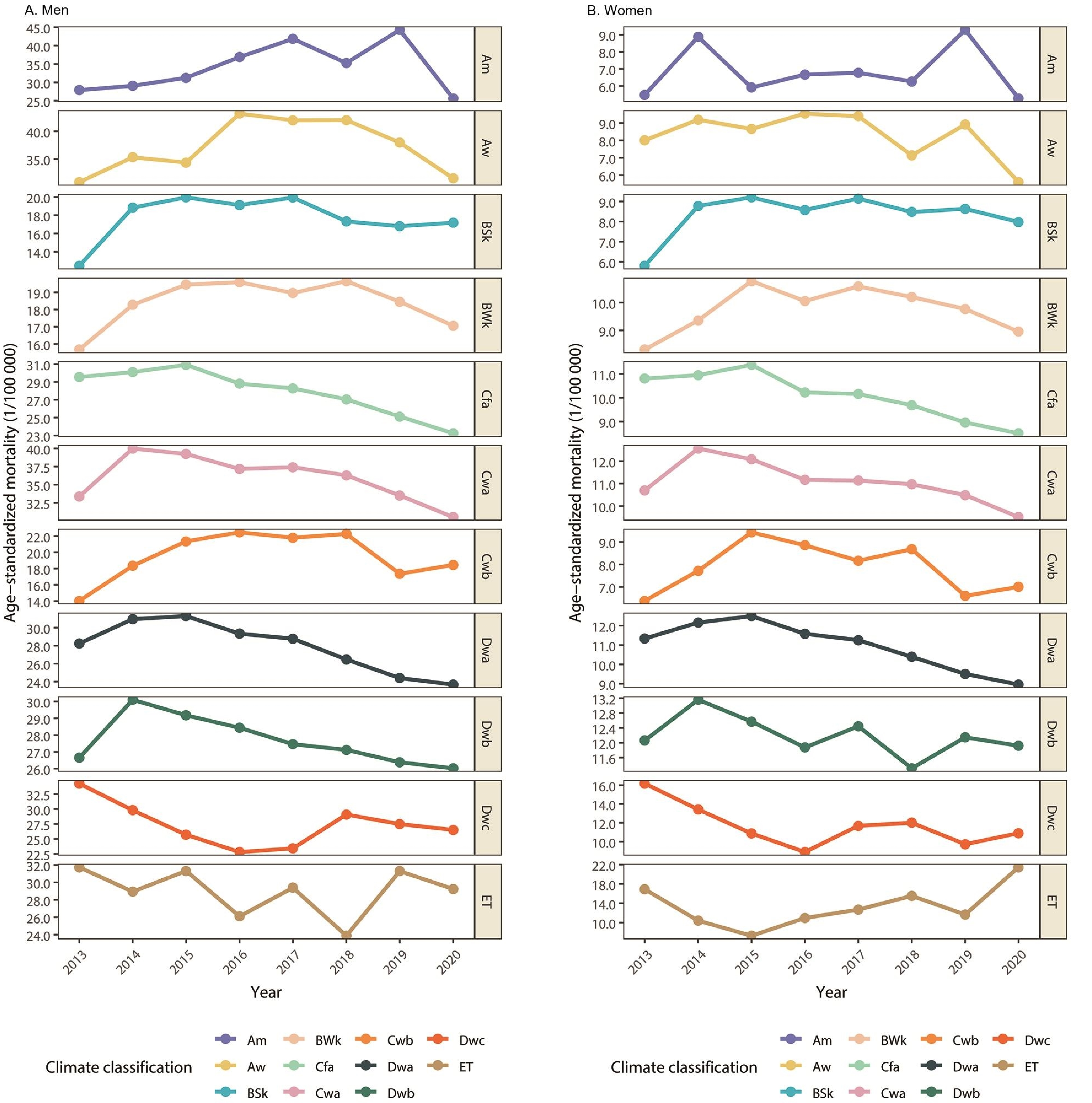
A. Men
B. Women
Am
Aw
BSk
BWk
Cfa
Cwa
Cwb
Dwa
Dwb
Dwc
ET
Age−standardized mortality (1/100 000)
Year
2013
2014
2015
2016
2017
2018
2019
2020
Climate classification
Am
Aw
BSk
BWk
Cfa
Cwa
Cwb
Dwa
Dwb
Dwc
ET
