# Supplementary material for: Liver Cancer Mortality Disparities at a Fine Scale Among Subpopulations in China: Nationwide Analysis of Spatial and Temporal Trends
Source: JMIR Public Health Surveill. 2024 Aug 8;10:e54967. doi: 10.2196/54967 (PMC11327839; doi:10.2196/54967)
Supplement: Multimedia Appendix 8 [file publichealth-v10-e54967-s008.docx]

**Multimedia Appendix 8.** Total years of life lost (10,000) of liver cancer in China (2013-2020).

|  |  | 2013 |  | 2014 |  | 2015 |  | 2016 |  | 2017 |  | 2018 |  | 2019 |  | 2020 |
| --- | --- | --- | --- | --- | --- | --- | --- | --- | --- | --- | --- | --- | --- | --- | --- | --- |
| China |  | 142.38 |  | 161.16 |  | 162.59 |  | 159.27 |  | 157.36 |  | 151.39 |  | 147.14 |  | 148.33 |
| Gender |  |  |  |  |  |  |  |  |  |  |  |  |  |  |  |  |
| Men |  | 101.48 |  | 115.42 |  | 115.94 |  | 113.53 |  | 112.03 |  | 107.44 |  | 104.63 |  | 104.60 |
| Women |  | 34.34 |  | 38.67 |  | 39.09 |  | 38.30 |  | 37.96 |  | 36.86 |  | 35.51 |  | 36.79 |
| Age |  |  |  |  |  |  |  |  |  |  |  |  |  |  |  |  |
| <35 |  | 6.38 |  | 7.06 |  | 6.89 |  | 6.90 |  | 6.80 |  | 6.09 |  | 5.98 |  | 5.70 |
| 35-49 |  | 42.42 |  | 46.74 |  | 44.68 |  | 42.00 |  | 40.07 |  | 36.64 |  | 33.85 |  | 31.99 |
| 50-65 |  | 59.18 |  | 68.39 |  | 69.78 |  | 69.05 |  | 68.14 |  | 65.66 |  | 63.27 |  | 63.80 |
| 65+ |  | 34.39 |  | 39.41 |  | 41.24 |  | 41.32 |  | 42.34 |  | 43.00 |  | 44.03 |  | 46.84 |
| Region |  |  |  |  |  |  |  |  |  |  |  |  |  |  |  |  |
| North |  | 9.95 |  | 11.85 |  | 11.69 |  | 12.23 |  | 12.21 |  | 11.11 |  | 10.77 |  | 12.41 |
| Northeast |  | 16.50 |  | 18.13 |  | 17.82 |  | 18.02 |  | 17.31 |  | 16.87 |  | 16.58 |  | 15.99 |
| East |  | 49.81 |  | 51.93 |  | 52.94 |  | 50.77 |  | 50.14 |  | 47.47 |  | 43.56 |  | 42.24 |
| Central |  | 21.08 |  | 26.31 |  | 26.51 |  | 25.33 |  | 25.53 |  | 24.71 |  | 25.20 |  | 25.47 |
| South |  | 18.53 |  | 23.65 |  | 23.58 |  | 23.73 |  | 23.57 |  | 22.94 |  | 22.73 |  | 22.33 |
| Southwest |  | 21.48 |  | 23.75 |  | 23.34 |  | 22.41 |  | 21.76 |  | 21.39 |  | 20.87 |  | 22.40 |
| Northwest |  | 5.04 |  | 5.99 |  | 6.71 |  | 6.78 |  | 6.84 |  | 6.90 |  | 7.43 |  | 7.49 |
| Climate  classification |  |  |  |  |  |  |  |  |  |  |  |  |  |  |  |  |
| Am |  | 0.20 |  | 0.24 |  | 0.25 |  | 0.28 |  | 0.32 |  | 0.26 |  | 0.32 |  | 0.23 |
| Aw |  | 1.03 |  | 1.22 |  | 0.99 |  | 1.27 |  | 1.24 |  | 1.07 |  | 1.17 |  | 1.14 |
| BWk |  | 1.89 |  | 2.27 |  | 2.49 |  | 2.54 |  | 2.42 |  | 2.52 |  | 2.69 |  | 2.62 |
| BSk |  | 4.11 |  | 6.30 |  | 6.49 |  | 6.67 |  | 7.09 |  | 6.48 |  | 6.65 |  | 7.57 |
| Cwa |  | 39.67 |  | 47.59 |  | 46.53 |  | 45.30 |  | 44.94 |  | 44.17 |  | 43.17 |  | 43.18 |
| Cwb |  | 1.63 |  | 2.11 |  | 2.52 |  | 2.66 |  | 2.41 |  | 2.72 |  | 2.14 |  | 2.37 |
| Cfa |  | 50.73 |  | 52.62 |  | 54.01 |  | 52.11 |  | 50.83 |  | 48.81 |  | 46.81 |  | 47.35 |
| Dwa |  | 38.19 |  | 43.66 |  | 43.99 |  | 43.19 |  | 43.06 |  | 40.16 |  | 38.82 |  | 38.38 |
| Dwb |  | 3.88 |  | 4.58 |  | 4.35 |  | 4.41 |  | 4.22 |  | 4.24 |  | 4.35 |  | 4.22 |
| Dwc |  | 0.77 |  | 0.70 |  | 0.60 |  | 0.54 |  | 0.56 |  | 0.70 |  | 0.64 |  | 0.68 |
| ET |  | 0.20 |  | 0.21 |  | 0.22 |  | 0.21 |  | 0.21 |  | 0.21 |  | 0.25 |  | 0.31 |
